# The activist health sciences librarian*

**DOI:** 10.5195/jmla.2020.859

**Published:** 2020-01-01

**Authors:** Gerald (Jerry) Perry

**Affiliations:** University of Arizona Libraries, University of Arizona, Tucson, AZ, jerryperry@email.arizona.edu

## Abstract

At the remove of 2019, it is hard for many to imagine the sense of apocalypse that was palpable throughout the gay community during the 1980s and much of the 1990s. My professional career was launched at the height of the acquired immunodeficiency syndrome (AIDS) pandemic, and at the time, saving lives through librarianship was my mission. This Janet Doe Lecture presents my personal story of activism and advocacy as a lens through which to consider the larger story of activism around social justice issues for the Medical Library Association, by groups such as the Relevant Issues Section, now the Social Justice Section, and by the work of past Doe Lecturers Rachael K. Anderson, AHIP, FMLA, and Gerald Oppenheimer. It is also the story of an association that has at times been deeply conflicted about the role of such activism in our niche of librarianship. With anchors in poetry and prose, this is a story of hope through justice, conveying a message of the essentialness of our work as librarians and health information professionals to the mission of saving lives.

## INTRODUCTION

Thank you, Elaine Russo Martin, FMLA, for that generous and kind introduction. It is an honor to be able to address my colleagues this morning for the 2019 Janet Doe Lecture. Today’s lecture comes on the heels of the outstanding 2016 lecture, “We Can Be Heroes: MLA’s Leadership Journey,” by M.J. Tooey, AHIP, FMLA, with its reference to my personal hero, David Bowie [1]. In 2017, we were riveted by the inspiring lecture, “Looking Inside Ourselves; A Culture of Kindness,” by Julia Sollenberger, AHIP, FMLA, which spoke directly to my spiritual heart [2]. Last year, Martin gave a brilliant presentation on “Social Justice and the Medical Librarian” [3].

When have we ever heard a Doe lecturer mention the groundbreaking work of Paulo Freiri and his seminal work, the *Pedagogy of the Oppressed*, with its challenge to us to break free of a colonial mindset around instruction [4]? In congratulating Martin on her stupendous performance, I had to share with her that every thought I had up to that point as to what I would speak on today was upended, as social justice was what I had hoped to base my lecture on. Ultimately, I decided I needed to be true to my authentic self, and so my lecture continues within the context of social justice but framed within the intersection of the personal, professional, and political.

All of our recent Doe lectures have touched on matters near and dear to my heart but, more importantly, near and dear to progressive visions of our profession as we continue ever forward in our collective journey of improving the quality of life through the application of accurate health information. My comments today will thus be centered on our values as an enabling profession and on activism as a necessary and explicit strategy.

Before advancing further, I would like to make a few acknowledgements to center myself and our collective presence here today. First, I would like to center myself as a gay, white, cis-gender male.

Our meeting site is the traditional home and trading site for several indigenous communities that flourished here until white settlers’ invasion and conquest, including the Potawatomi, Miami, and Illinois Nations [5]. It is necessary for us who do the important work of advancing a health improvement agenda that we are conscious of history, just as we must be conscious of the social determinants of health that so deeply inform the need for our work.

I would also like to acknowledge today my racism. As a white man, it is simply a given that I experience the privileges that come with being white and male in an American culture that both explicitly and implicitly celebrates white supremacy. I center my racism today as a call to action to all other white people in the room to raise our collective awareness of the need every day to interrupt unquestioned racism and, as a white person, to be responsible and accountable every day for what our privilege means historically. To fail in these acts is to be complicit in the racism that has so negatively impacted people of color.

Now, I am fairly certain that a good number of you are looking at your devices or eyeing the door, looking to make an exit rather than endure a politically correct harangue from an old white liberal with a microphone. I am not going to walk back from my centering comments, because they represent me, and I take pride in bringing my whole self to my work.

But I also pride myself on being open to others’ experiences, and I welcome everyone in this room today, especially those who disagree with me. I ask for your indulgence on this journey into identity and how it has informed both my own anger and hope, and perhaps the anger and hope of others, in the role of health sciences librarian activist.

I ask you to think about your place in the world, as who you are and where you live are important to your identity, to the whole of you that I hope you bring to your work. For this reason, today’s lecture includes images of my beloved Sonoran Desert home. For the time we have together, I hope you will consider the backdrop of images not as a distraction from the words, but rather to give weight to what it means to be fully present. For that presence, as Sollenberger reminded us, is a tool for expressing our values of compassion and kindness, which underlie the mission-based work that we perform [6]. Being present is an essential part of the context that lets us do our very best work and is, therefore, a useful tool.

One of the tools that I find effective for helping me to be more present in time and space is the reading of poetry and personal essays. And so, rather than harangue you with sports analogies as I did in 2012, I shall instead harangue you today with poems and quotes from essays that I hope will illustrate my Doe message. I will argue that each of you here in this hall today is an activist and that, in 2019, it is essential that you own that role. By the example of a former mentor, Rachael K. Anderson, AHIP, FMLA, MLA president in 1997 and your Doe lecturer in 1989 [7]; by the example of Gerald Oppenheimer, founding president of the Association of Academic Health Sciences Libraries (AAHSL) and your 1988 Doe lecturer [8]; and by my own activism, I hope to convince you of the historically relevant and necessary work of activism in the profession. I hope to describe and frame our association’s complex and at times problematic relationship with the “polis,” being the political components of the environment in which we work [9]. I hope to convince you of the need to embrace engagement with the polis and to claim our place in community, framed by that which we are uniquely qualified to represent. I hope to entreat you to not only take a stand, but to proactively advocate for positive change and for our association to be the front edge of the wedge as we force open structures and systems that prevent us as librarians from having the quality of impact that I believe we must have in order to advance equity, fairness, and improvements in health status for all.

Somewhere back there, I mentioned poetry. For those who have not met her, please let me introduce to you to fellow librarian, Audre Lorde [10]. Audre Lorde is known primarily as a Black, lesbian, feminist poet and essayist, but she was also an academic and public librarian. While she may not have coined the phrase, Lorde conceptualized for us the notion of intersectionality, whereby we have come to understand that the personal experience of class, race, sexual orientation, age, religion, disability, and gender, among other characteristics, do not exist separately but, in fact, are interdependent [11].

Lorde’s writing largely focused on her experiences of intersectionality, on Black female identity, and on civil rights and social justice themes. In her essay on “Uses of Anger,” Lorde wrote:

Every woman has a well-stocked arsenal of anger potentially useful against those oppressions, personal and institutional, which brought that anger into being. Focused with precision, it can become a powerful source of energy serving progress and change. And when I speak of change, I do not mean a simple switch of positions or a temporary lessening of tensions, nor the ability to smile or feel good. I am speaking of a basic and radical alteration in those assumptions underlining our lives. [12]

Today, I am asking you to hold in your mind Lorde’s framing of the uses of anger—especially anger over inequity, discrimination, and oppression—as we consider the history of activism in our profession. I ask this in full recognition of the discomfort that the notion of anger inspires. We as a community, as humans, tend to avoid that which makes us uncomfortable, and we strongly avoid expressions of anger as they may be seen as unprofessional. Lorde was indeed angry, and she was a technical master of the word, and she used her mastery to direct her anger assiduously. By raising up Lorde’s example of the uses of anger, I am not encouraging incivility, but rather her precision, focus, and intention in service. I do not know if Anderson’s or Oppenheimer’s activism was spawned, let alone encouraged, by anger. But I know they were technical masters of the word. I can rightfully attest that my own activism was indeed spawned by anger, that which arose in light of the acquired immunodeficiency syndrome (AIDS) pandemic.

At this remove in 2019, it may be hard to recall the sense of apocalypse that was endemic in the lesbian, bisexual, gay, trans, questioning (LGBTQ) community in the late 1980s and early 1990s. Some of you may be too young to remember that time, or perhaps your connections to the queer community were such that you simply did not know the collective and individual toll that human immunodeficiency virus (HIV)/AIDS was exacting. Or, like me, you may remember too well the untimely funerals, the panic when a condom broke, the distraught pacing while you waited for your test results.

In January of 2019, the Fox Network broadcast a staged presentation of the musical *Rent* on national television [13]. As many of you know, the plot line revolves around the lives of a community of “bohemians” living in the East Village of New York City, several of whom are infected with HIV, during the era of which I now speak. A lyric to the chorus of the song, “What You Own,” goes

And when you’re dying in America
at the end of the millennium
you’re not alone
I’m not alone. 
[14]

That is a chorus that every person in this room who lived through and lost friends to the pandemic will recognize, before the antiretroviral cocktail of 1995 gave hope back to a devastated community. Therein are the paradoxes of fear and hope, rage and resilience, despair and comfort that *Rent* at its debut gave time and space for us to feel, now over twenty years ago. To borrow a motif from Puccini’s *La Boheme*, upon which *Rent* is based, the experience of HIV/AIDS in the gay community in the mid-1980s up to the mid-1990s was the spark that lit the fire of my anger, and it was the spark that drove me into activism and a career in health sciences librarianship.

In the mid-1980s I was a club kid living in Buffalo, working days at the Buffalo General Hospital’s Aaron Health Sciences Library, under the direction of my first mentor, Wentsing Liu. Work offered me the opportunity to go to the University of Buffalo to get a master of library science (MLS) degree.

A chief medical resident at Buffalo General became a friend, and he guessed my sexual orientation without our ever talking about it. He gently suggested I take a look at the alarming headlines about the new gay cancer in the medical newspapers I was checking in.

Nearing the end of my MLS coursework, I had the opportunity to take an independent study and spent a semester researching and compiling an annotated bibliography on what was then known about AIDS. That independent study saved my life.

My bibliography completed, I soon graduated and was recruited to the University of Illinois at Chicago’s Library of the Health Sciences on the near west side. Moving to Chicago gave me the opportunity to fully embrace a gay identity in a city far from home, family, and the worry of censure. It also introduced me to AIDS activism, ACT UP, later Queer Nation, and the politics of direct action.

In Chicago, I immersed myself in the literature of AIDS and queer fiction. I joined a writers’ group, published poetry and short stories, and performed short compositions. I imagined a life in the arts but thankfully never quit my day job. Instead, I decided to bring more of myself into my work and began to make connections between the role of the librarian in helping not only health practitioners, but also consumers in using information to save and transform lives. I started to see that our work as health sciences librarians was a weapon. Woody Guthrie famously referred to his guitar as a weapon that kills fascists [15]. At the time, I saw my work as a weapon against HIV/AIDS.

I was also keen to disrupt the notion of what a health sciences librarian should look like, what they should do, wear, and say. I was intellectually inspired at the time by the Beats and especially Lawrence Ferlinghetti’s brilliant “A Coney Island of the Mind” [16] and his poems from *Pictures of the Gone World* [17]. Ferlinghetti turned 100 years old this year, and to him, I wish mazel tov. To quote from poem 6:

Truth is not the secret of a few
yet
you would maybe think so
the way some
librarians
and cultural ambassadors and
especially museum directors
act

You’d think they had a corner
on it
the way they
walk around shaking
their high heads 
[18].

Disrupting that image was part of my agenda.

At the University of Illinois at Chicago, I was introduced to academic medical librarianship at a time when that library was being integrated with the campus’s Main Library. For me, the academy was anything but the “Gone World.” It was energizing, exciting, maddeningly complex but also inspiring. I met my second mentor, the great science bibliographer H. Robert Malinowsky, who took me under his wing. Together, we contracted with Oryx Press to edit the *AIDS Information Sourcebook*, a reference book including a chronology of the disease, a directory of support organizations across the country, and a bibliography [19]. The 1988 edition ran eighty-five pages. Our goal was to connect information seekers and providers with information that we believed could save their lives. We wrote the following in the introduction to that first edition:

The need for education about AIDS is so crucial that words fail to adequately address the problem. There can never be too many resources for AIDS education. We all need to be educated because AIDS is everyone’s concern...Compassion empowered with knowledge can achieve miracles. Short of a miracle, education is the only way to prevent the spread of AIDS and save lives. [20]

Malinowsky and I eventually edited 3 editions of our reference book, with the third and final edition published in 1991. Topping out at 300 pages, the book is a testament to those pre-“AIDS cocktail” years, when accurate health information about HIV and AIDS was the only defense. In the “Introduction” to the third edition, we recognized the progress that had been made and the challenges yet to come. To quote:

In the past, the group at highest risk for contracting the AIDS-causing Human Immunodeficiency Virus (HIV) has been the homosexual population. In fact, many of the resources that exist today are based upon the efforts established by and for the gay community to provide education in an attempt to control the spread of AIDS…The toll has been overwhelming, both in terms of human life and in medical costs. However, the effort has paid off. Due to education campaigns in the gay community, the incidence of new cases among gay men in this country is at its lowest level in years and is steadily decreasing. However, other groups are now at a higher risk of contracting HIV than ever before, namely intravenous drug users and blacks and Hispanics. Efforts to provide education to these groups have been hampered by many barriers including cultural factors, language and religion. [21]

With hindsight, this quote strikes me as naïve and deeply white and male centered. The real barriers were racism, sexism, and classism, and as we all know, those barriers are still with us today.

By the time the last edition of *AIDS Information Sourcebook* came out, I had taken a new position as an education librarian at Rush University. There, I found a deeply nurturing culture under the leadership of progressive Director Trudy Gardner, my third mentor the wonderful Chris Frank, and a community of supportive queer librarians. It was a politically informed workspace, and the queers began to agitate. In 1990, we asked what the Medical Library Association (MLA) was doing to prevent association and chapter meetings from being held in cities that were passing anti-LGBTQ ordinances or actively fighting queer cultural events, such as when the Midwest Chapter was considering meeting in Ohio in 1991. We were responding to an infamous skirmish in the culture wars, when Ohio’s Hamilton County prosecutors decided to file obscenity charges against the Cincinnati Contemporary Arts Center’s director for his museum’s hosting of a controversial Robert Mapplethorpe photo exhibit [22]. My Rush colleagues and I stepped into those battles and pushed MLA to take sides. We asked headquarters leadership why we should be asked to travel to cities where our gayness made us unwelcome.

Looking back, I wonder now at our boldness and fierce independence. I saw myself then as responsible for myself, in a deeply radical way. Ever the fan of Verlaine, Rimbaud, Ferlinghetti, and Ginsburg, it was not a stretch for me to also see the future liberation of self in the poetry of the high priestess of punk, Patti Smith, who infamously wrote in the poem, “Oath”:

Jesus died for somebody’s sins but not mine
melting in a pot of thieves
wild card up my sleeve
thick heart of stone
my sins my own
I engrave my own palm
sweet black X
Adam placed no hex on me
I embrace Eve and take full responsibility. 
[23]

Saving lives through librarianship, thusly, took on a new guise in my mind, militant and willing to disrupt and transgress, wearing the mantel of righteous anger over lack of equity and inclusion, albeit from an LGBTQ perspective. Absent was my recognition that other communities and especially persons of color lived with inequity all of the time. Such was my white, privileged, racist, male-centered outlook. Nonetheless, I persisted.

In 1993, the MLA annual meeting was held in Chicago, and for that meeting, my Rush colleagues and I took it upon ourselves to create an alternative, queer-friendly guide to Chicago, including gay bars and restaurants; we even included a bathhouse. We wanted to do something that was positive and demonstrated to MLA’s LBTGQ members that they were welcome in our hometown. We used our connections with the local hospitality committee to ensure that copies of *Q++*, as we called it, were distributed at the meeting hotel. While some MLA members were appalled, others were delighted, and folks back in the library who were not attending the meeting received panicked phone calls from the Hospitality Committee asking for yet more copies.

Some of those *Q++* copies made it into the hands of other LGBTQ-plus colleagues, and through word of mouth, we connected. By the 1994 annual meeting in San Antonio, we had organized and were working to secure approval through MLA’s Section Council to establish the Lesbian, Gay and Bisexual Special Interest Group (SIG). Transgender would be added later. The proudest day of my work life happened the day I sent an email to the MEDLIB-L email discussion list announcing our efforts to form the SIG. In so doing, I essentially came out to everyone in our professional niche, including any potential future boss. For some members, I became the gay face of MLA.

There was a backlash. I received death threats and deeply unsettling phone calls from anonymous colleagues who wanted to express their disgust toward me and the other queers in MLA. There was also a lot of politicking, as members of the core team that organized around the SIG petition worked to make sure that the Section Council vote to approve the interest group was based on an equitable assessment of our group’s mission and not rooted in anti-gay bias. We met in San Antonio as an informal group, and it was both a happy and heartbreaking moment, as members from across the organization came together and shared their stories of challenges and fear, but also joy that they had found connection with others like them in MLA, all accompanied by a lot of tears.

Our fear of having an inequitable vote was such that I insisted on speaking with the MLA Board of Directors to make the case that, if the vote was not successful, it would need to be reviewed so that we could be sure bias did not play a role. I can still recall that conversation, sitting down with the MLA Board in a Chicago hotel meeting room and the shock and surprise around the table as I described that highly emotional San Antonio meeting.

Fortunately, our SIG was approved, and our cluster of queer activists soon found a welcoming connection with the Relevant Issues Section. Truth-to-tell, this was not by happenstance, as I had been active in the Relevant Issues Section since attending my first MLA meeting, in Portland, Oregon, in 1987. The LGBTQ SIG has since its inception been closely linked with the Relevant Issues Section, largely because we share a similar public health frame of reference regarding our work, one that recognizes that social conditions are inevitably linked to the lived experience of health for individuals and communities.

The history of the Relevant Issues Section, which in 2018 changed its name to the Social Justice Section, is worthy of our attention, as this section has long been considered the social conscience of MLA. James Anderson, a leader of the section who helped with its 2018 name transition, recently reminded me that the very concept of MLA embracing a social justice mission continues to be controversial, given push-back received from some association members who, during the discussion about the name change, did not see the relevance of social justice to the profession.

Established by petition in 1972 under the leadership of University of Pennsylvania librarian Jerome Rauch, the Relevant Issues Section came about at a time of social upheaval against the war in Vietnam and across Southeast Asia. Oppenheimer’s brilliant Doe lecture from 1988, “Domus or Polis? The Location of Values,” framed the Relevant Issue Section’s emergence from the debate in MLA about the necessity of considering the broader societal context in which the association operated, not necessarily from a concern based in the social determinants of health but rather from a worldview that posited the necessity of engagement with the broader society in which we live and work [24]. Oppenheimer’s lecture examined the gradual development in MLA, and I quote, “of a consciousness of its place in society as an organization of professionals holding certain values, the reflection of those values in association attitudes, and their expression in what may broadly be called political activity” [25].

As Oppenheimer reported, at the 1970 MLA annual meeting, sixty members submitted at the meeting’s open convention the following motion:

We move that the following statement be forwarded as a letter…to President Richard M. Nixon as our official resolution of the Medical Library Association…We urgently request the reordering of national priorities away from their emphasis on war and military actions towards peace and health service programs…we demand an immediate cessation of military actions in Laos and Cambodia and the prompt withdrawal of U.S. armed forces from Vietnam. [26]

The parliamentarian declared that the motion was out of order due to not being compatible with the association’s bylaws. Our MLA president at the time, Elliott Morse, supported the position of the parliamentarian. According to Oppenheimer, his motivation was rooted as much in a concern about the motion as the adoption of “an expanded set of values” and the potential for division among the membership in light of the contentious political climate at the time [27].

As Oppenheimer noted in his Doe lecture, “the narrow conception of relevance could not be maintained much longer. Individual members had kept alive the interest of those who favored participation of the association, as it was put, ‘in the affairs of the real world’” [28]. Thus, from the conflict over the Vietnam War was the Relevant Issues Section, then called the MLA Relevance Group, born, with the cohort developing and circulating a National Priorities and Peace Petition at the 1972 annual meeting. According to Oppenheimer:

The turning point occurred at the 1975 annual meeting in Cleveland when the Relevance Group brought to the general business meeting a lengthier Petition on National Priorities and Health. After pointing out the low priorities of health related programs for federal funding, the petition concluded: “Therefore, be it resolved that the Medical Library Association strongly urges both the Congress and the President of the United States to reconsider priorities and reappropriate funds towards a renewed emphasis on finding solutions to the health problems of this nation.” [29]

The floor discussion framed the core issue at hand: a member stated that it was not the business of the association to enter the political arena. According to Oppenheimer, “Erich Meyerhoff responded that, on the contrary, whenever our interests are involved, we have a right ‘to speak up as an association which has given this function to the Legislative Committee’” [30].

We have seen in the years since a commitment to legislative action, evinced by MLA efforts through committee work and in partnership with AAHSL, to advocate for the prioritization of funding that supports the National Institutes of Health, including the National Library of Medicine, and for legislation that advances the cause of health-related research and information access [31]. Meyerhoff was a leader of the Relevance Group and was one of my mentors into the workings of MLA when I joined the Relevant Issues Section in 1986.

The Relevant Issues Section has also been deeply engaged in driving our association to step up to leadership by adopting policies and positions that take moral stances on social and political issues that evince our values as information providers. In 1983, the section sponsored an annual meeting resolution calling on MLA to support the concept of a bilateral freeze on the development, production, and deployment of nuclear weapons and delivery systems. The resolution passed [32].

In 1986, the Relevant Issues Section advanced to our board an anti-apartheid resolution that was approved [33]. In 1987, the section sponsored an annual meeting resolution on the AIDS crisis, which read:

*Whereas*, we are an organization committed to the improvement of health; and *whereas*, the AIDS epidemic threatens the lives of many citizens of our country; *therefore be it resolved* that the members of the Medical Library Association will identify and employ innovative strategies to disseminate information concerning AIDS to all members of the health care team, to patients, and to the public. [34]

This resolution too was passed by the membership in attendance. All this consciousness-raising has not been without its consequences.

The degree to which MLA provides a welcome to what Oppenheimer called the polis, including political and social issues, has, as I have previously noted, been contentious, and it continues to be so. The degree to which our association welcomes diversity, and especially people of color, is linked to the ongoing domus versus polis debate that, though not articulated in those terms, first arose for MLA at about the time leading up to the Second World War, not surprisingly when you consider the galvanizing impact of the war effort and the need to set aside divisions for the good of the whole [35]. During times of challenge, we often hear and abide the call to come together, to be resilient in defiance of the times. The other side of that coin is the call to eschew that which reflects our individuality, seeing difference as a distraction from the core mission. That tension between welcoming inclusivity and the desire for solidarity often has negative consequences, especially for those who are a minority in the collective.

The great Harlem Renaissance master of poetry and verse, Langston Hughes, understood that tension and countered through explicit recognition of that which is universal for those oppressed. He wrote the clarion-call poem, “In Explanation of Our Times,” from which I quote:

The folks with no titles in front of their names
all over the world
are raring up and talking back
to the folks called Mister.
You say you thought everybody was called Mister?
No, son, not everybody.
In Dixie, often they don’t call Negroes Mister.
In China before what happened
they had no intention of calling coolies Mister.
Dixie to Singapore, Cape Town to Hong Kong
the Misters won’t call lots of other folks Mister.

Hughes continues,

Hell, no! It’s time to talk back now!
History says it’s time
and the radio, too, foggy with propaganda
that says a mouthful
and don’t mean half it says—

but it is true anyhow:
LIBERTY!
FREEDOM!
DEMOCRACY!
True anyhow no matter how many
Liars use those words.

Mister Hughes concludes,

So, naturally, there’s trouble
in these our times
because of people with no titles
in front of their names. 
[36]

It is long past time to discuss the trouble our association has had with its contextualization of race. As described in a two-part series in 2004 and 2005 on “Race and Librarianship” [37, 38] by the *Journal of the Medical Library Association*’s then History Editor Carolyn E. Lipscomb, AHIP, FMLA, “MLA lagged behind other professional associations in integrating its membership. Not until the end of a decade of discussion in the 1930s were black libraries admitted” [39]. As Lipscomb describes, MLA policy at the time afforded voting rights to libraries rather than individual members. Approvals to join our association as a library were made by the Membership Committee, which solicited new members, and the Executive Committee, which approved the applications. Libraries who wished to join were required to meet both collection and staffing standards, not the least of which was the ability to participate in the Exchange, the service whereby member institutions posted and shared lists of journals in excess and in demand, and agreed to trade to fill holdings gaps. The primary reason given at the time for excluding Black libraries was the perception that, based on their small scale, these libraries would be unable to participate in the Exchange.

The other reason, apparently articulated primarily through correspondence between members of the Executive Committee, were social, rooted in the fear of dividing and alienating members of what was then a small and homogenously white community. According to Lipscomb:

Throughout the 1930s, the libraries of Meharry Medical College and Howard University School of Medicine attempted to join MLA but were excluded. The Executive Committee was described as having voted on the question three times and having discussed it at every meeting for nine years. [40]

Letters requesting admission from the leadership of both libraries and their supporters were submitted. According to Lipscomb, “Janet Doe, as secretary of MLA, distributed the letters to the Executive Committee and polled them on two questions: ‘a) shall Meharry Medical College Library be admitted? b) If so, shall other Negro libraries be advised of our change of policy?’” [41]. The vote of the Executive Committee was positive to admit Meharry but split equally as to the change of policy to admit Black libraries to membership, even if they met the requisite standards. This refusal was based in fear of social disruption of MLA meetings, should Black librarians attend meetings and become part of the group.

Doe supported the admission of Meharry and Howard and, at the time, stated, “There are bound to be some complications, but we sincerely hope they won’t be as serious as if the principle of impartial treatment were not accepted” [42].

In hindsight, would we consider Doe to be a disruptor of the sort described previously by Langston Hughes? Was she an activist, in the sense of that word as we have come to know it in the new millennium? These are, of course, rhetorical questions.

In 2019, we know Doe for her progressive vision of medical librarianship and for being an exemplar of the role of women in advancing the organization and advancing health information as an essential asset to medical progress. Was she an activist? I believe so.

Anderson, our Doe lecturer in 1989, noted the long-term effects of racial prejudice and gender-based bias on both librarianship and our association. In her lecture, Anderson, who was my boss and mentor in the late 1990s, stated:

The deeply felt, negative, personal convictions of several individuals who were among the associations’ most active members and leaders for another generation betoken a continuing inhospitable climate for recruiting minorities to the field…We can ask ourselves, with regard to both major societal issues—gender and race—to what extent we are now reaping the consequences of our profession’s past inhospitality to women and minorities. [43]

Anderson’s lecture investigated the question of who “has or has not entered the profession of medical librarianship” [44]. She noted the lag in numbers of women in administrative roles and the salary gaps they experienced when compared with men. In her lecture, she stated:

My presentation today is unabashedly influenced by the problems of recruiting staff that many of us have been experiencing in recent years…We cannot ignore the strong influence that societal attitudes toward gender and race have exerted on library staffing patterns…I think libraries have not recovered from their profound effects, and we continue to experience their negative consequences. [45]

Anderson framed her review of the profession as a call to open an avenue for research “leading to a general redefinition of professional expectations and practice in light of current and future requirements” [46]. Her assessment embraced the question of MLA’s readiness for gender and racial equity. Her Doe lecture was also a clarion call to health sciences librarianship to adjust our thinking around gender, especially, in light of the needs of the profession to evolve away from past stereotypical notions of role, capacity, and ambition.

Citing data from the mid-1980s, Anderson noted increases in the numbers of women ascending to directorships of libraries, along with the persistence of salary gaps between women and men. The fiscal year 2017 *Annual Statistics of Medical School Libraries in the United States and Canada*, published by AAHSL, noted that men continue to earn as much as $5,500 more per year than women for the same position level, region, experience, medical school type, and position type [47]. Yes, this problem persists nearly 30 years after Anderson’s Doe lecture.

As many of us in this room who do diversity, equity, and inclusion (DEI) work already know, deciding to take up the burden of this labor is akin to a life sentence. By this, I mean that DEI work is “forever” work. Fighting for equity is something that we will have to do until people stop “othering” each other. That does not suggest we should give up hope. Having hope despite the odds is our moral and ethical imperative.

Established in 2017, MLA empaneled a task force to “Evaluate and improve practices as they relate to diversity and inclusion within MLA” and has directed the team to consider MLA’s foundational documents including mission, vision, values, and code of ethics; to consider association programming; to address the formulation of position statements; to encourage members to engage in difficult discussions around equity and justice; and to encourage diverse individuals to participate in association leadership [48].

Just as it is forever work, so too is DEI work episodic in nature. Experience has taught us that whenever a particularly heinous national outrage or crisis occurs, people remember that equity matters. We saw grassroots interest in gender equity spike over twenty-five years ago in response to Anita Hill’s testimony at Supreme Court Justice Clarence Thomas’s confirmation hearings [49]. Nationally, we are experiencing a spike of sorts as so many of us grapple with the division in our nation over the Trump presidency. I would hazard that MLA’s establishment of its Diversity and Inclusion Task Force is emblematic of a current episodic moment. I am not here to chastise our association for waiting until 2017 to establish a DEI task force, but I will note that it is about time. MLA is not alone: AAHSL, for which I presently serve on the Board of Directors, only recently established its own DEI committee [50].

Linking MLA’s and AAHSL’s recent epiphanies around equity to Anderson’s clarion-call Doe lecture and to the socially informed gaze that Oppenheimer exhorted us to embrace in his own pivotal lecture, I would incite the active, or latent, activist in you to consider who is in the room today. Is there a person of color sitting at your table? Are we as an association as diverse in our membership as we wish to be? Are we willing to overcome the resistance that will continue to arise whenever we recognize when and where we have fallen short in our vision of an equitable association?

In 2013, as immediate past-president, I chaired MLA’s Nominating Committee and worked with a terrific team of elected individuals to develop a roster of candidates for the Board of Directors and the president-elect position. As a result of that experience, I drafted an internal white paper for the board about our committee’s experience in trying to put together a diverse roster. One of the challenges we experienced was the myth that our president must have board experience in order to be electable. This rationale is grounded in very reasonable logic: that MLA Board experience prepares one for the challenges of the presidency. I challenged that myth, because I believe that a combination of other leadership experiences in MLA can prepare one to be successful. This mattered to me because the myth of prior board experience is one of the reasons, I believe, it took us until now to have a Black president of MLA [51]. So few people of color have served on the board, and if we assume that board experience is requisite, then the odds were not in favor of our ever electing a person of color to our highest leadership position. I was, thus, thrilled by the election of Beverly Murphy, AHIP, FMLA, as our first Black president, and at the same time, I know that as long as this myth persists, we must continue to advance persons of color as MLA Board candidates if we are to achieve the goal of consistently equitable leadership for MLA.

Another myth that haunts our association is the chimera of the domus versus polis binary that Oppenheimer addressed in his Doe lecture and which I have referenced several times in this talk. When I helped to establish the LGBTQ SIG, members of MLA who were opposed told me that by establishing such a SIG, I was contributing to division in the association, the logic being that organizing around a subgroup identity would result in SIG allegiance as opposed to allegiance to the shared goals of the association, resulting in disunity. This same thinking reared its head when the Relevant Issues Section recently changed its name to the Social Justice Section. This same thinking informed the delay in admitting Meharry Medical College to our association.

With all due respect and courtesy to those who believe and invest in this myth, I must return to and remind us all of the value that comes when we welcome all identities into our association and when we embrace all aspects of ourselves. Our work is not a zero-sum game, nor is there a limit to the sorts of work we can do as health information professionals. The giants of our profession, and among them we must include Janet Doe as well as Gerry Oppenheimer and Rachael Anderson, have always exhorted us to expand our horizons, cast a wider gaze, and embrace the new in order to remain a viable community of practice.

Attendant to the notion that to celebrate the contributions one’s lived experience brings to our work is divisive is the myth that MLA is somehow not a political organization. We know, however, that this is not true. Were it so, we would not have a Joint MLA/AAHSL Legislative Task Force that lobbies Congress through Capitol Hill visits and would not create and disseminate position statements, nor would MLA sign on to advocacy letters in concert with peer organizations to advance what we think are the right and necessary policies to enable the health sciences.

My “Jerry Perry” opinion is that what truly lies at the core of these myths is the very real human worry about having difficult conversations around privilege. Why is it that some of us would deny that social justice is something that the profession needs to care about, when the public health schools we support teach a model of disease impact and burden called the social determinants of health? Why is it that there continue to be pay inequities between male and female wage earners in health sciences librarianship? Why it is that there are so few people of color in this room today? These questions are about the intersection when the personal becomes political and where the political has a place in the professional work we do. These questions are why I described in this lecture my own personal story leading up to my activism. This is justice work, and librarianship IS in fact justice work, as Elaine Martin so eloquently argued at last year’s lecture [52].

My hope for the work of the MLA diversity, inclusion, and equity group is that through their efforts we as an association will build resilience for the hard conversations we need to have in order to make MLA the inclusive and welcoming organization that we all wish it to be.

For me, the welcoming began when I found mentors who did not merely tolerate but actively encouraged my activism. People like Wentsing Liu, Bob Malinowsky, Trudy Gardner, Chris Frank, Rachael Anderson, Gary Freiburger, AHIP, Rick B. Forsman, AHIP, FMLA, and so many more, who recognized that my activism was rooted in my personal identity, and that when I claimed equity for my personhood in my work, in my scholarship, and in my service, it was a political act executed at the professional level.

To deny anyone’s complete personhood is simply not acceptable. As we as a profession advocate for positive health outcomes through the work we do to bring the right piece of information to the right person in the right format when and where it is needed, we must recognize the activism required of each of us to achieve those ends. Just as the hospital librarian must be an activist around the role of evidence-based information and data to ensure patient safety and fewer readmissions in their accountable-care organization, so too must the academic librarian be an activist around equitable and paywall-free barriers to health information to support research, scholarship, and community engagement.

Our work requires of us the commitments of conscience to advocate for our clients, for the patients they serve, and for the communities in which we work and that bear the burden of illness and disease. Our work requires of us the commitment to advocate for truth in medicine and health care, the preservation and veracity of the intellectual record, and the critique of that record as the basis for advancing new knowledge. Our work requires of us the commitment to advocate for equity in access to information, equity in the opportunity to participate in research, and equity in how the results of research are applied.

Seen through these commitments, ours is truly a profession motivated by the quest for justice. Seen from these perspectives, each of you in the room today is an activist. We are all engaged in the forever work of social justice, on behalf of our trainees and patients.

You have stayed with me across nearly an hour, and I want you to know how indebted I am to you for listening. I also wish to acknowledge my debt of gratitude to my partner Garry Forger. I would like to acknowledge my incredible team at the University of Arizona Health Sciences Library, including my Associate Director Annabelle Nuñez and the administrative staff of Mikel Bates and Curt Stewart.

Thank you, Elaine, for the terrific introduction, thank you to the past Doe lecturers who set a bar too high, and thank you to MLA, my professional association home for nearly thirty-five years. I am a decidedly imperfect choice for your Doe lecturer, but one who cares deeply about our work. A truth about myself is that I am always looking for redemption. And so, I am going to leave the last words of this lecture to Leonard Cohen, the great Canadian poet and musician:

Like a bird on the wire
Like a drunk in a midnight choir
I have tried in my way to be free.
Like a worm on a hook
Like a knight from some old-fashioned book
I have saved all my ribbons for thee.
If I, if I have been unkind
I hope that you can just let it go by.
If I, if I have been untrue
I hope you know it was never to you.

For like a baby, stillborn
Like a beast with his horn
I have torn everyone who reached out for me.
But I swear by this song
And by all that I have done wrong
I will make it all up to thee.
I saw a beggar leaning on his wooden crutch
He said to me, “You must not ask for so much.”
And a pretty woman leaning in her darkened door
She cried to me, “Hey, why not ask for more?”

Oh, like a bird on the wire
Like a drunk in a midnight choir
I have tried in my way to be free. 
[53]

## SUPPLEMENTAL FILE

Appendix Slides from the 2019 Janet Doe Lecture

## 

**Gerald (Jerry) Perry, MLS, AHIP, FMLA**, jerryperry@email.arizona.edu, University of Arizona Libraries, University of Arizona, Tucson, AZ
