# Supplementary material for: The activist health sciences librarian
Source: J Med Libr Assoc. 2020 Jan 1;108(1):5–16. doi: 10.5195/jmla.2020.859 (PMC6920003; doi:10.5195/jmla.2020.859)
Supplement: Appendix [file jmla-108-5-s001.pdf]

JMLA Journal of the Medical Library Association

Supplemental content to
J Med Libr Assoc. Jan;108(1):dx.doi.org/10.5195/jmla.2020.859
www.mlanet.org
© Perry 2020

# "The Activist Health Sciences Librarian"

Jerry Perry, MLS, AHIP, FMLA

*Janet Doe Lecturer*

May 6, 2019

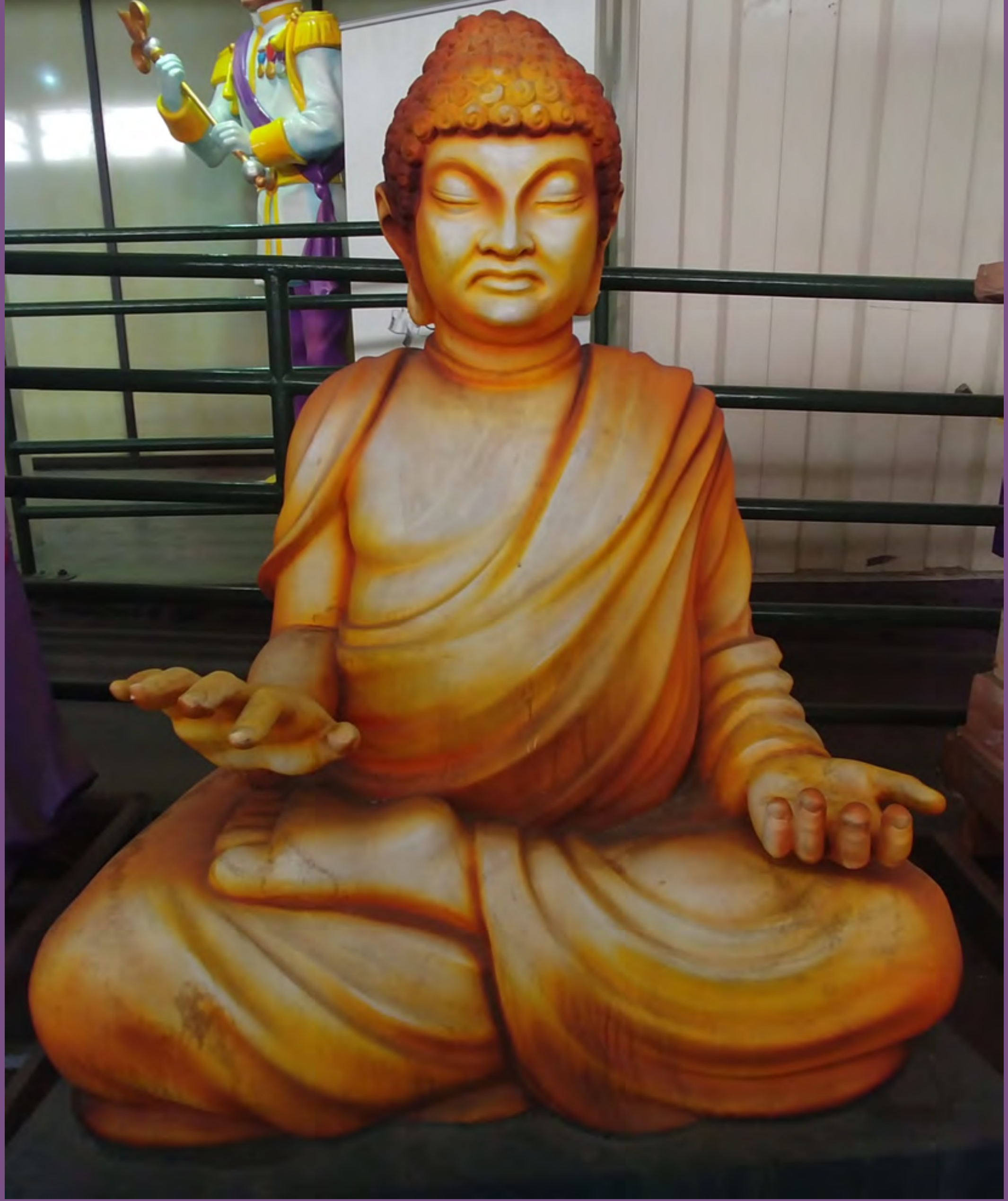

Buddha float, Mardi Gras World, New Orleans

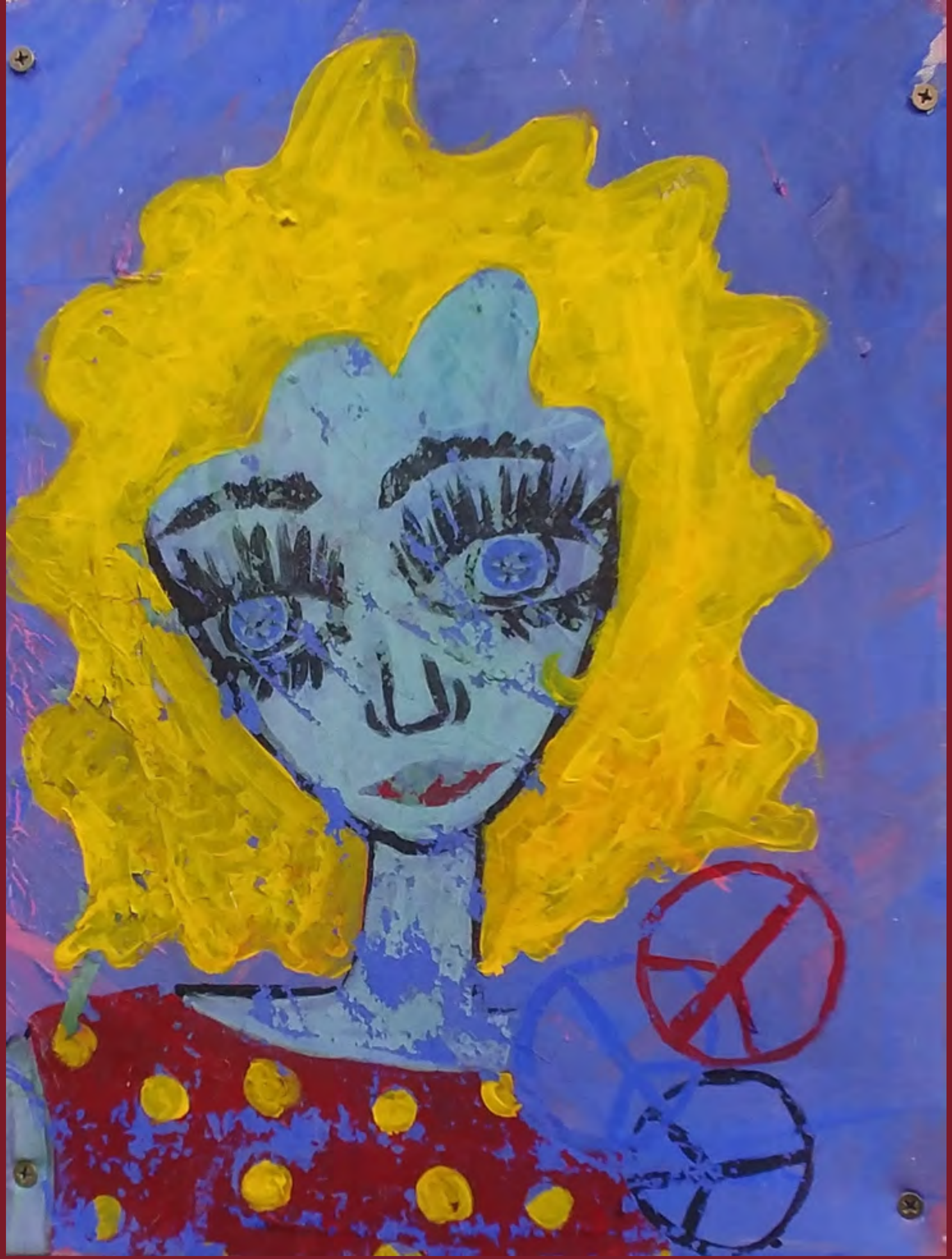

Street art, Bisbee, Arizona

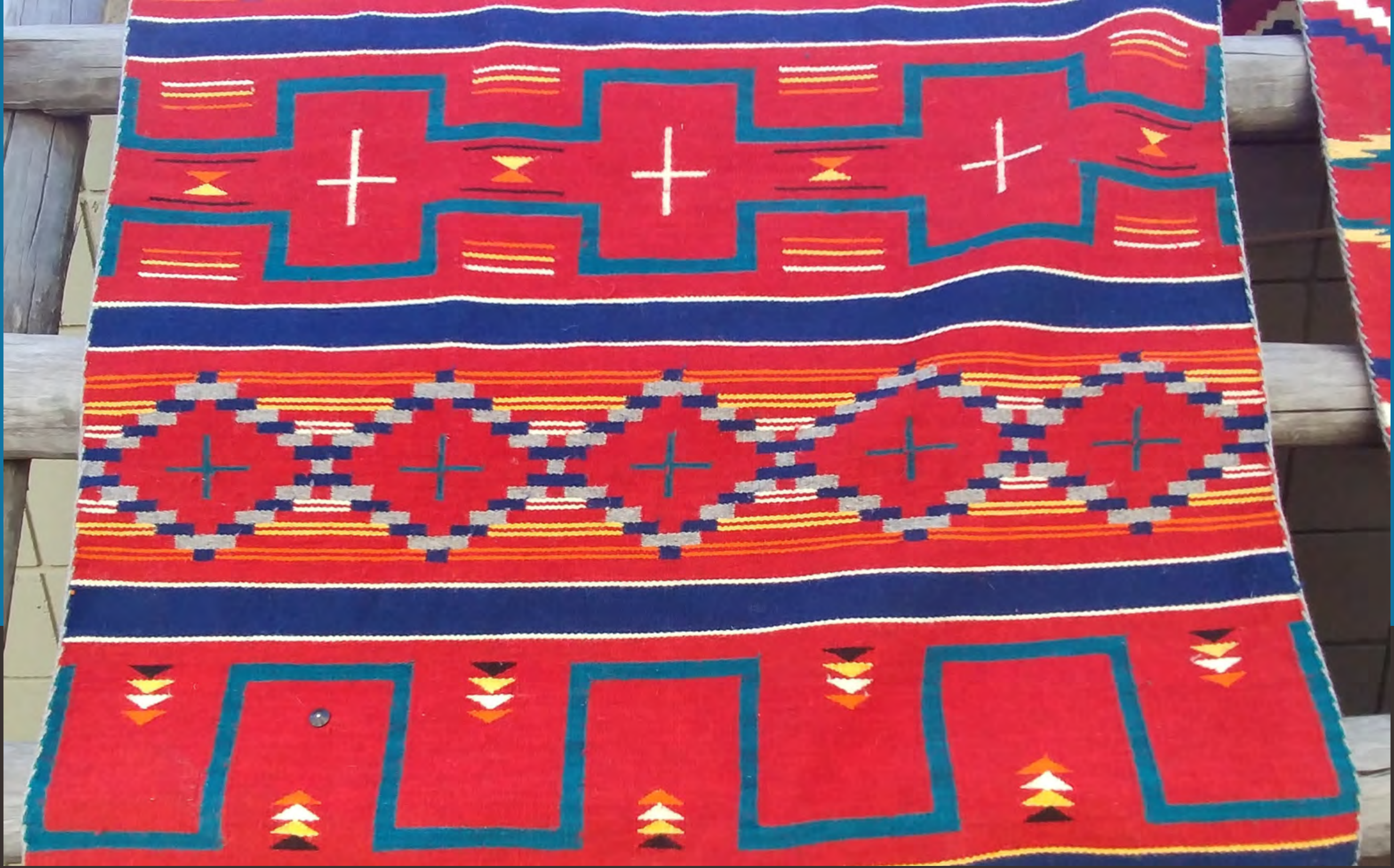

Contemporary Navajo blanket

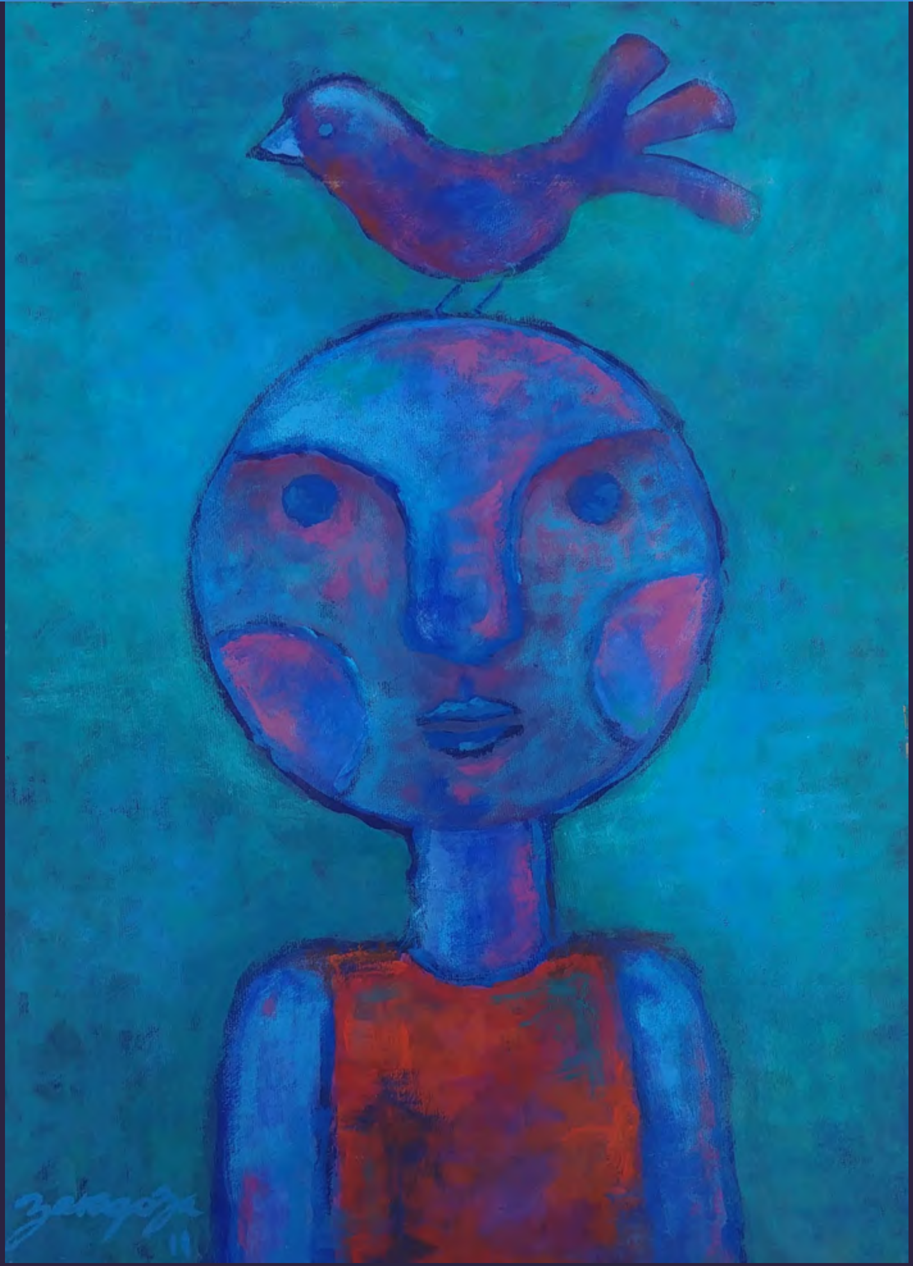

Thrift shop art, unknown Mexican artist

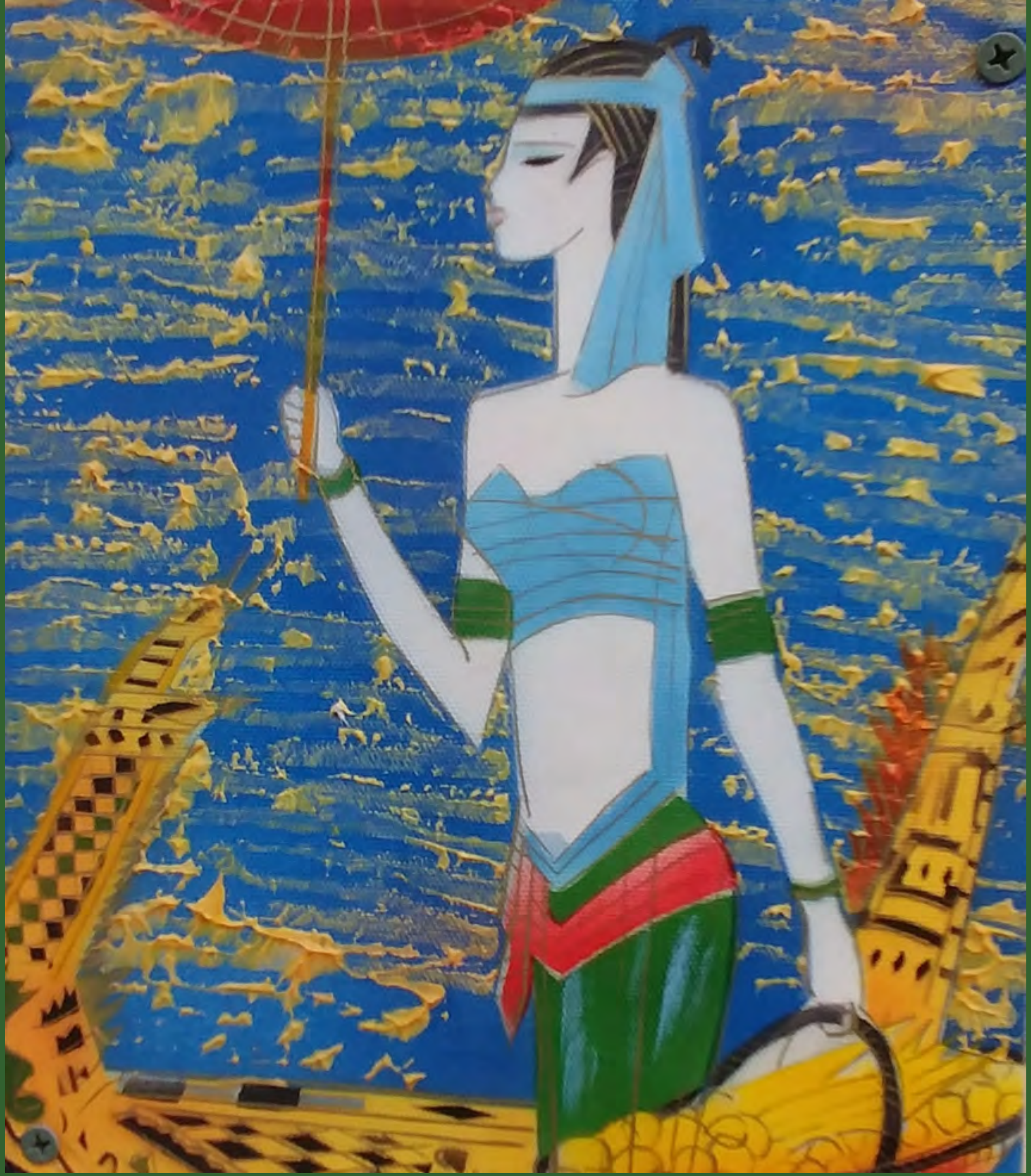

Street art, Bisbee, Arizona

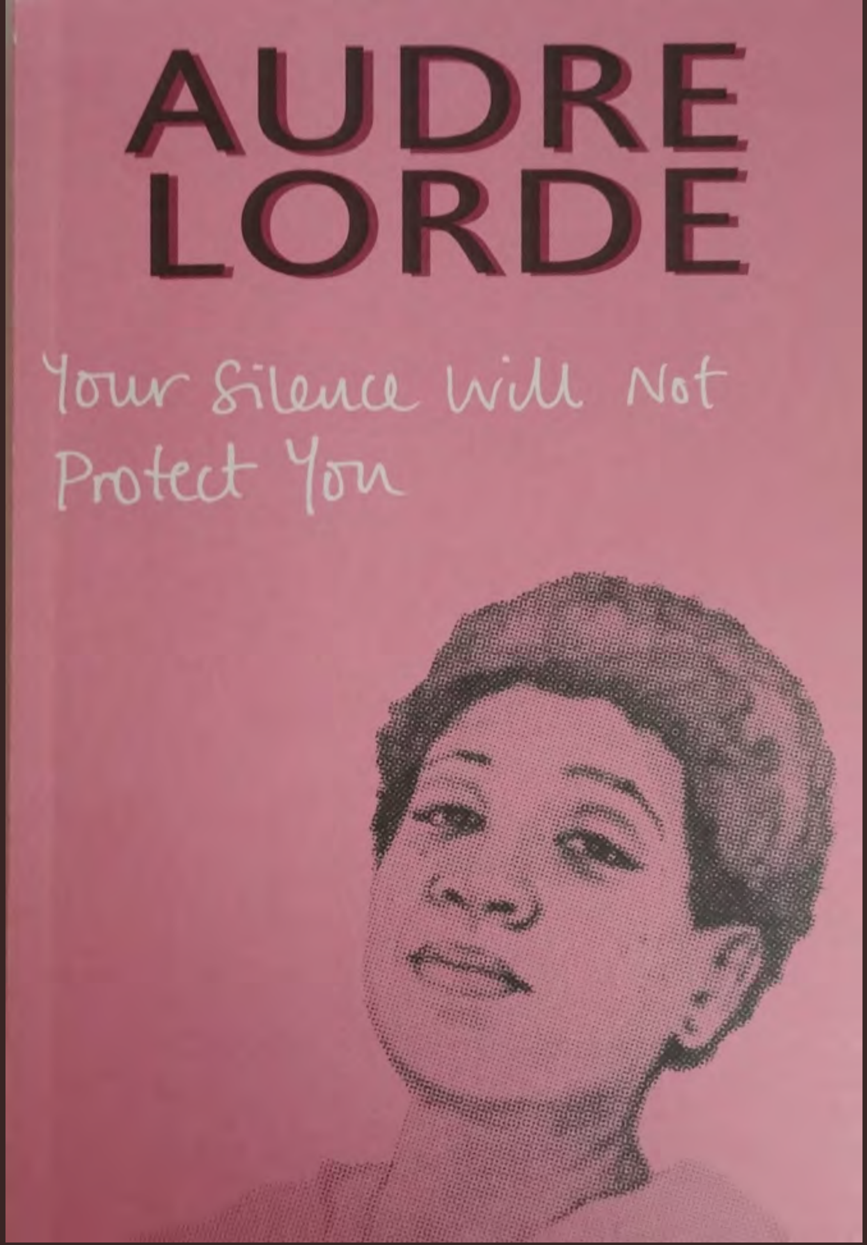


Audre Lorde, *Your Silence Will Not Protect You*.
London, UK: Silver Press; 2017

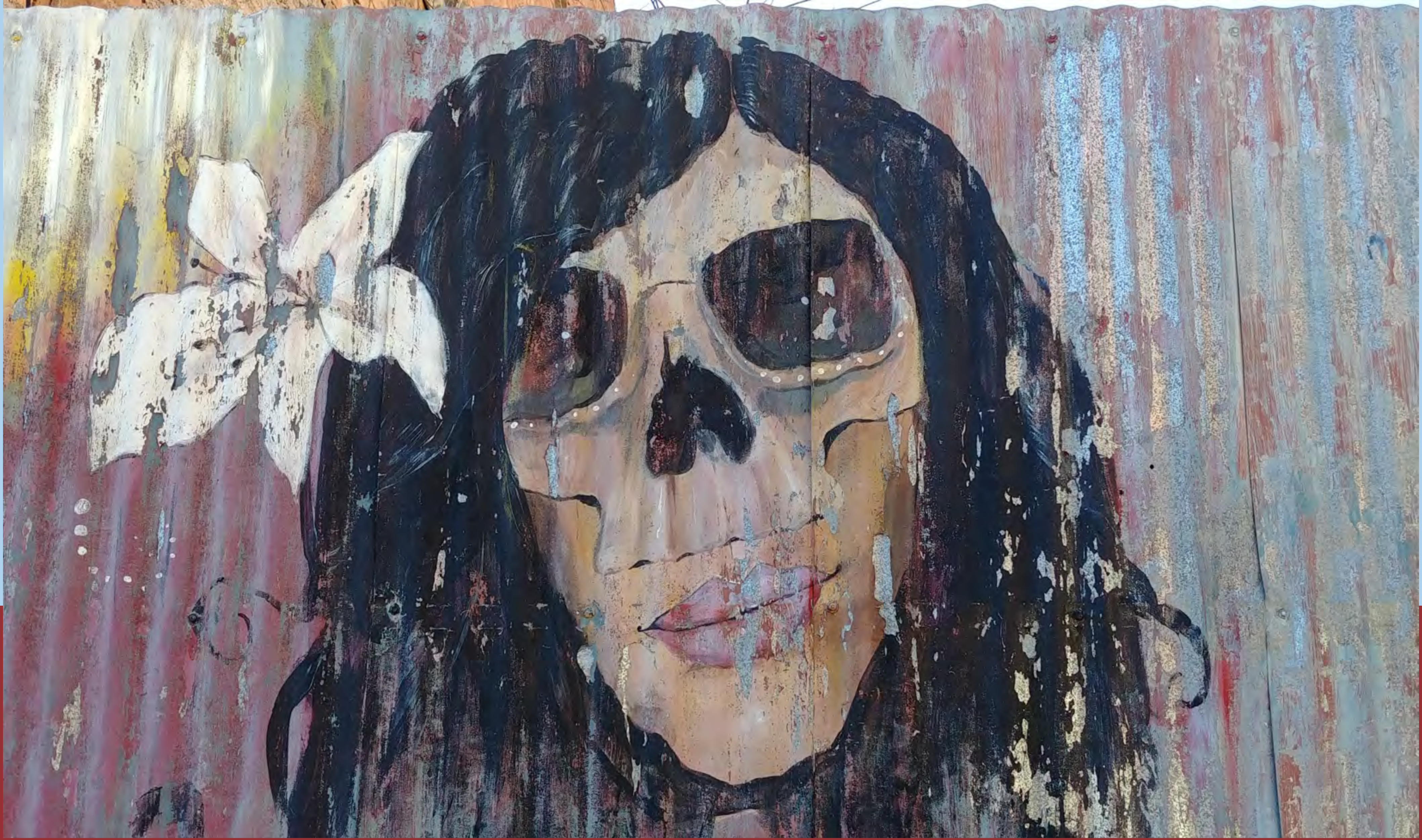

Skeleton woman, Bisbee, Arizona

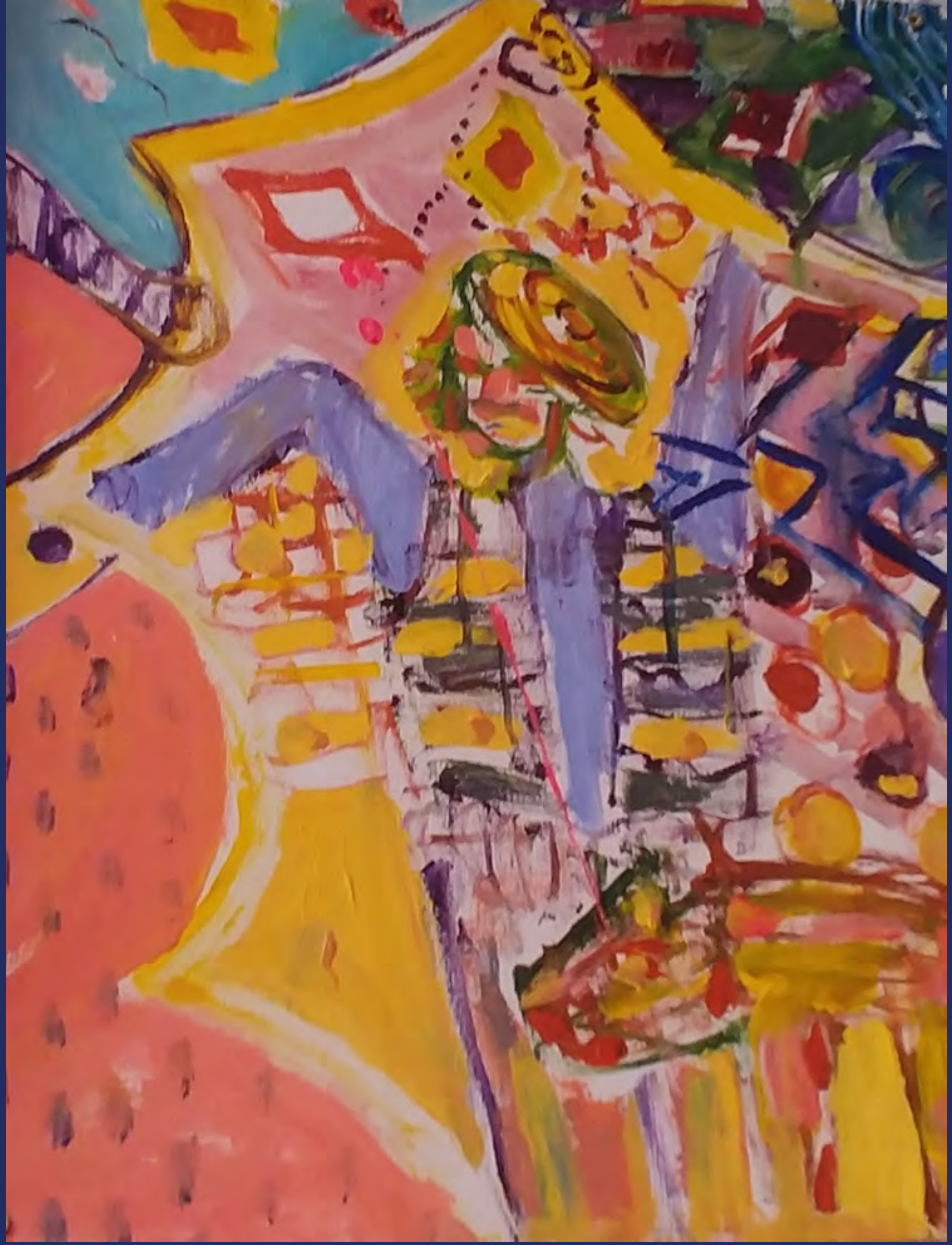

Street art, Bisbee, Arizona

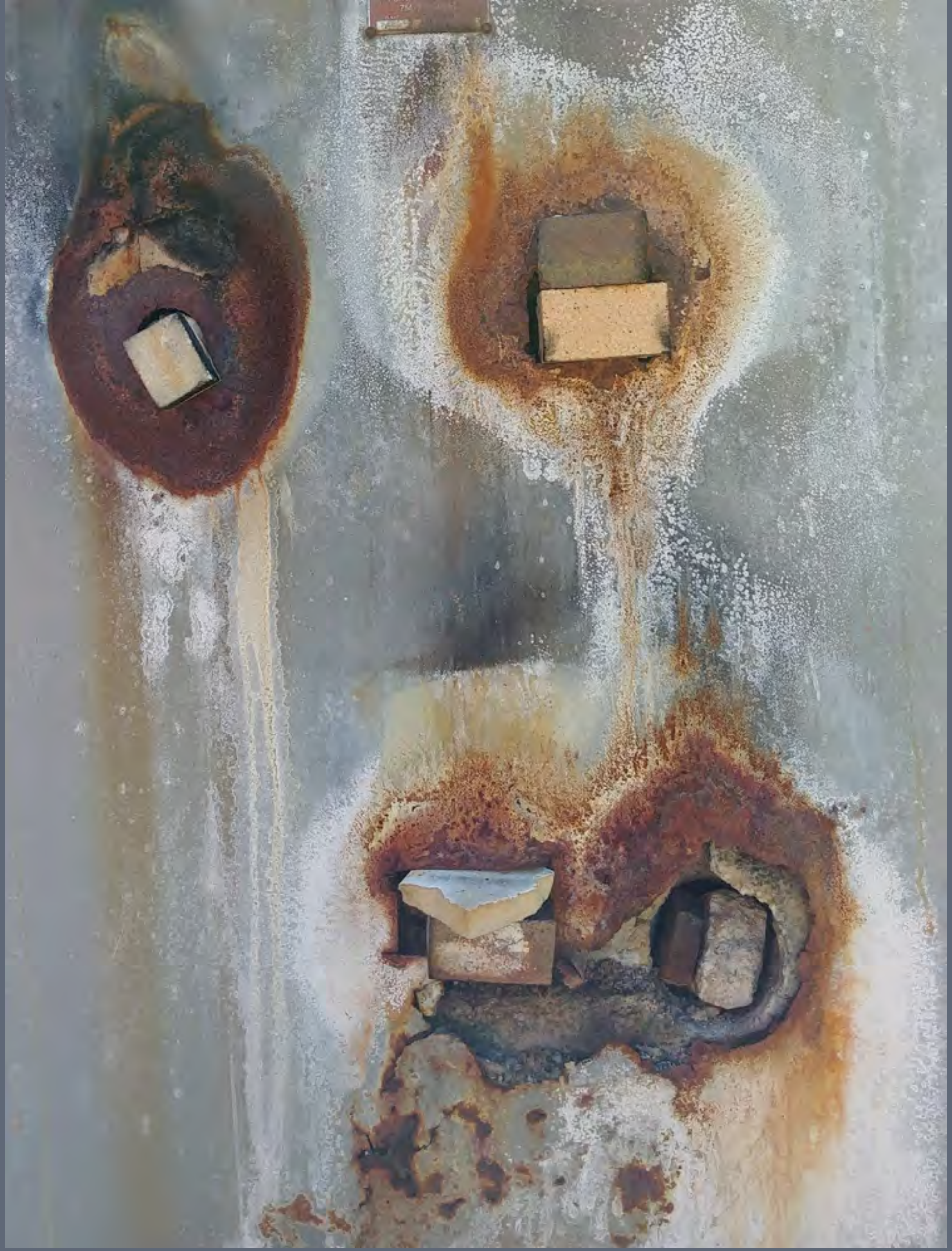

Rust, Scottsdale, Arizona

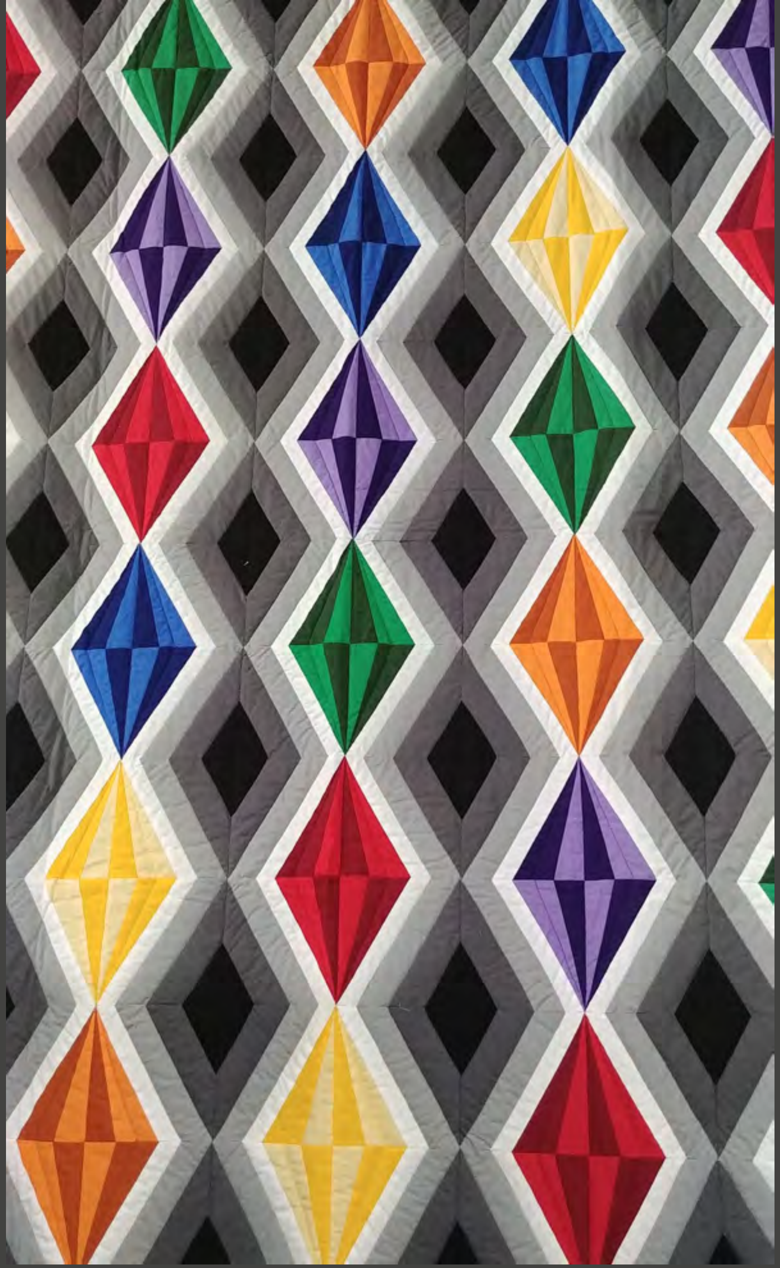

Quilt, Arizona State Fair 2018

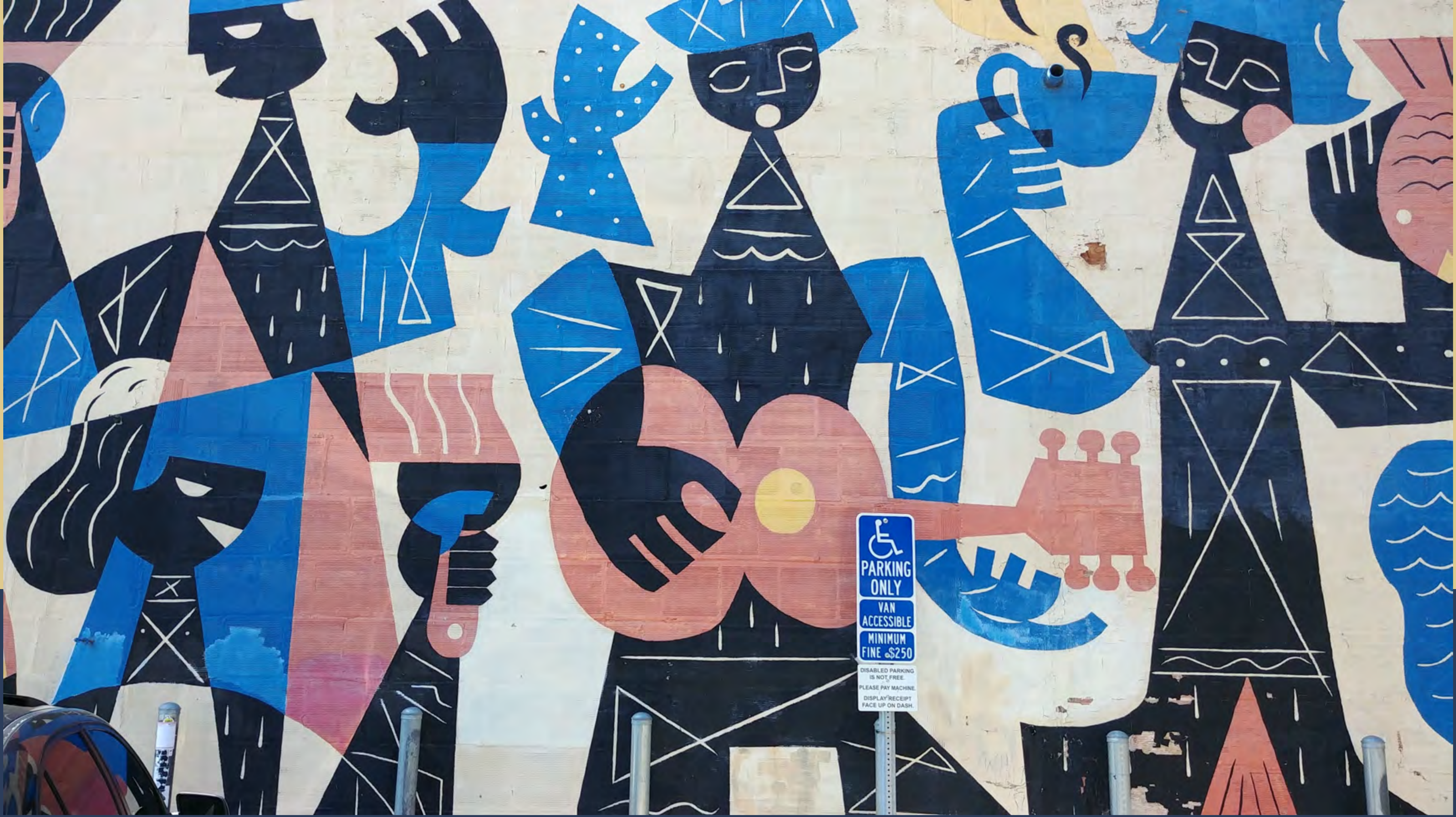


Mural, but where?

ISSN 1044-2138

UNIVERSITY OF ARIZONA
39001030387354

# AIDS INFORMATION SOURCEBOOK

Third Edition 1991–92

H. ROBERT MALINOWSKY
GERALD J. PERRY

ORYX PRESS

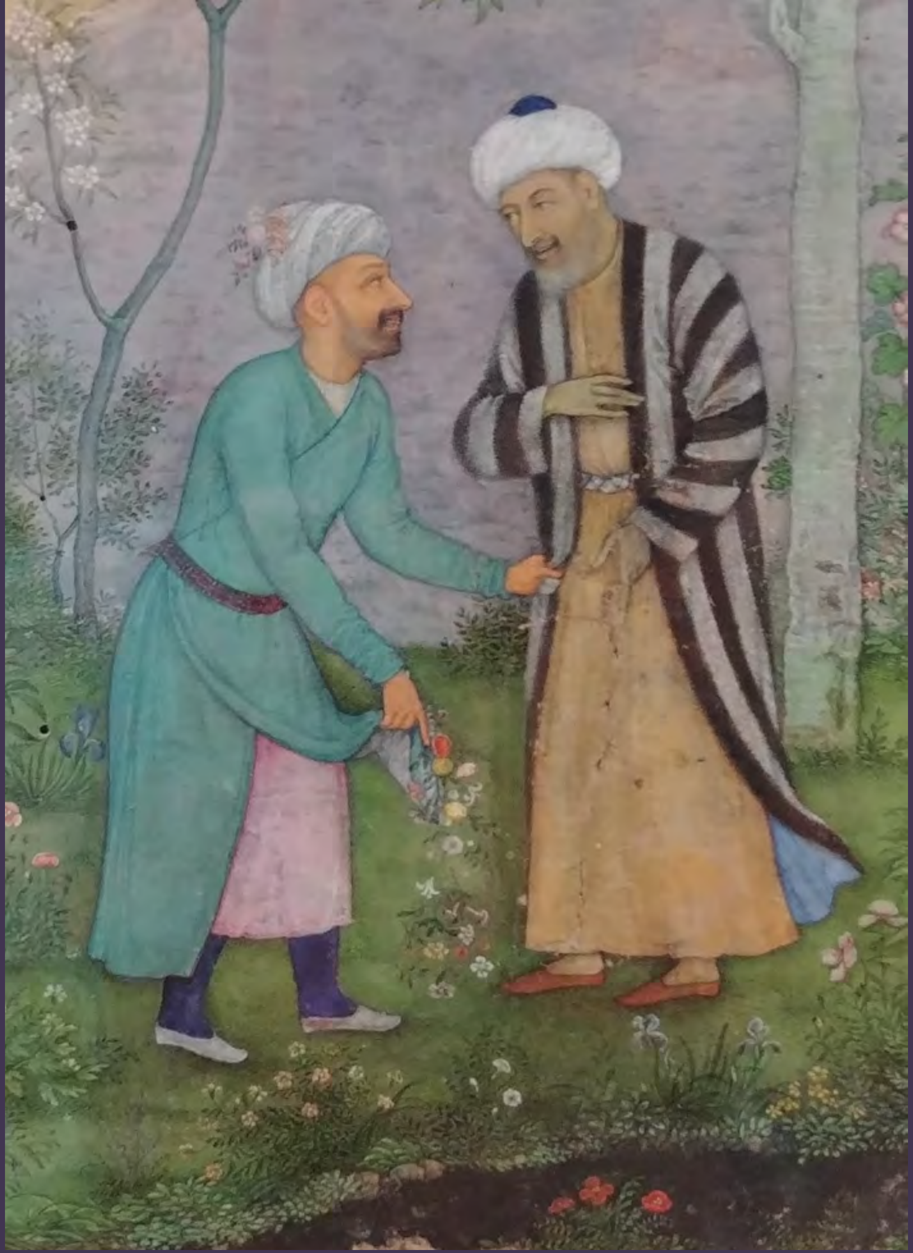

Persian art, Freer Gallery, Smithsonian Museum

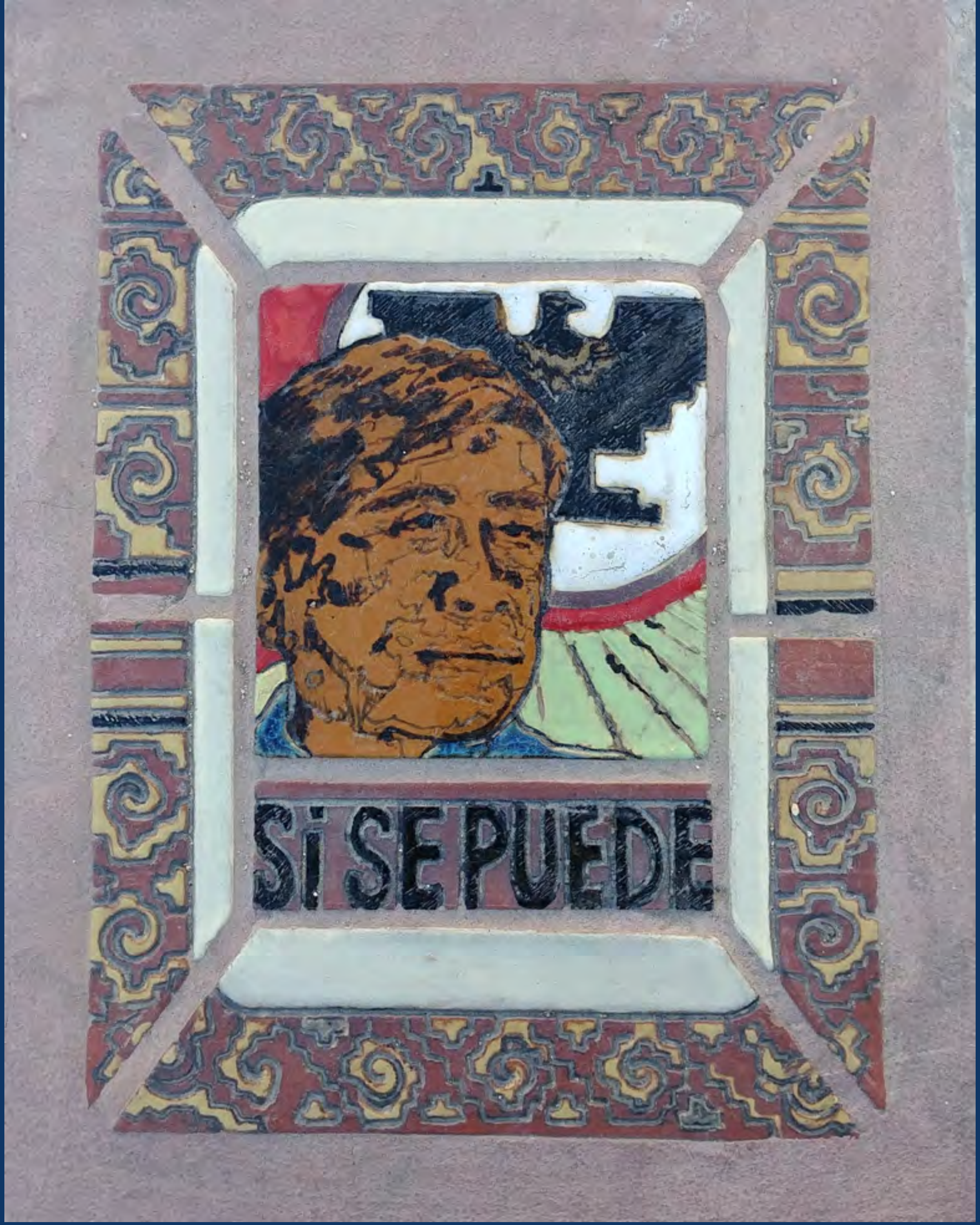


Cesar Chavez tile, Mercado San Augustin, Tucson

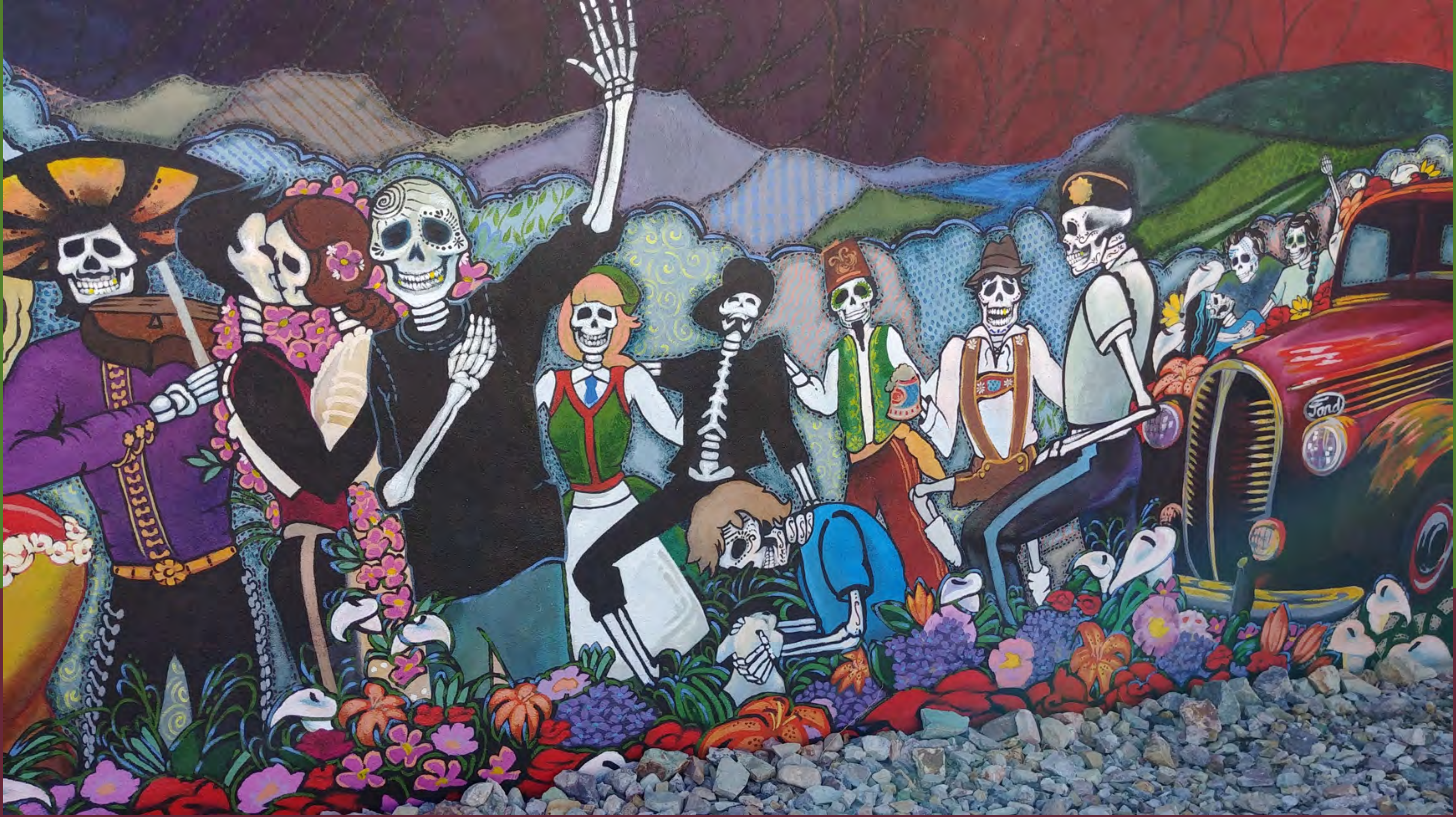


Dia de los Muertos mural, Why, Arizona

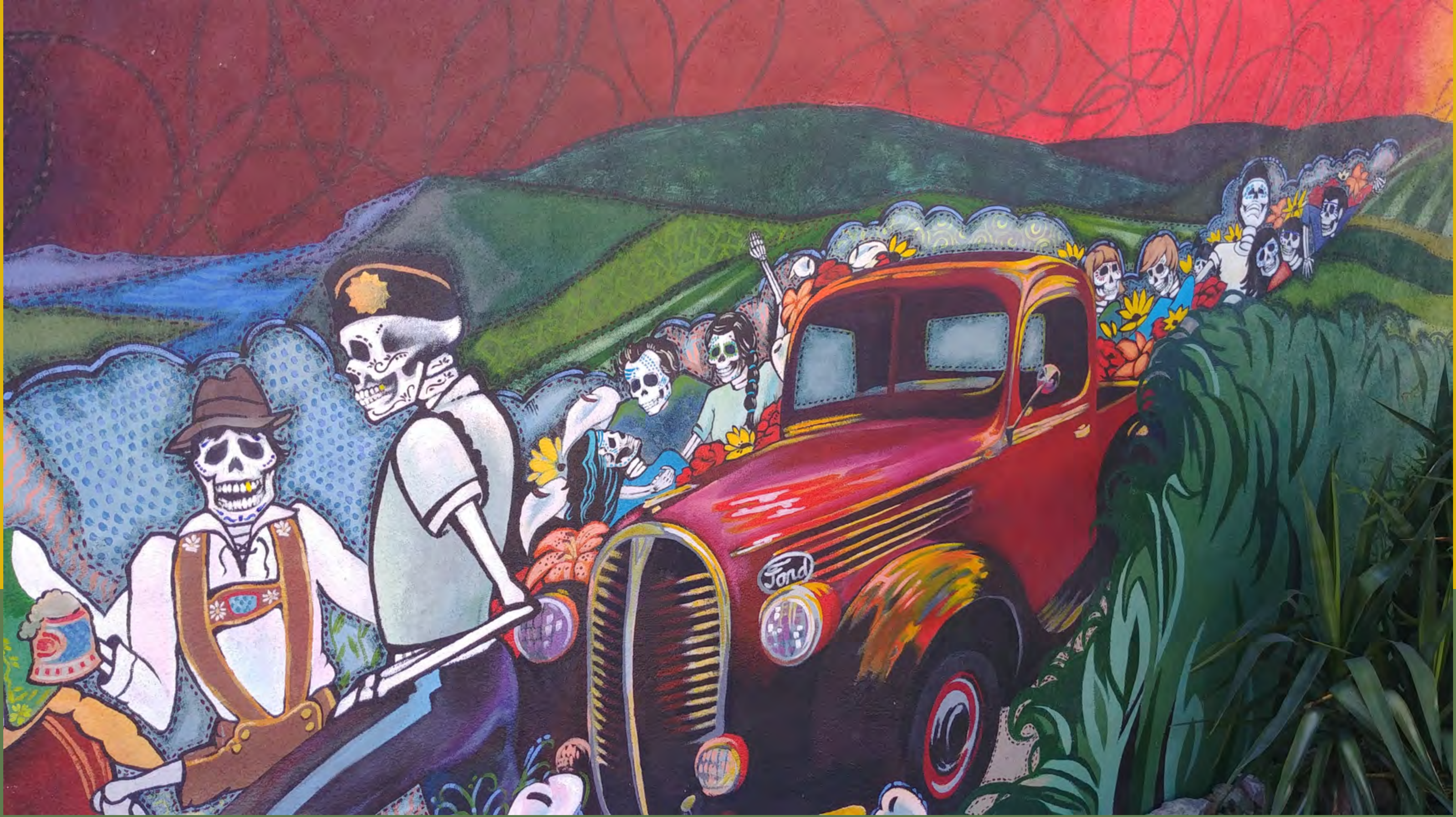


Dia de los Muertos mural, Why, Arizona

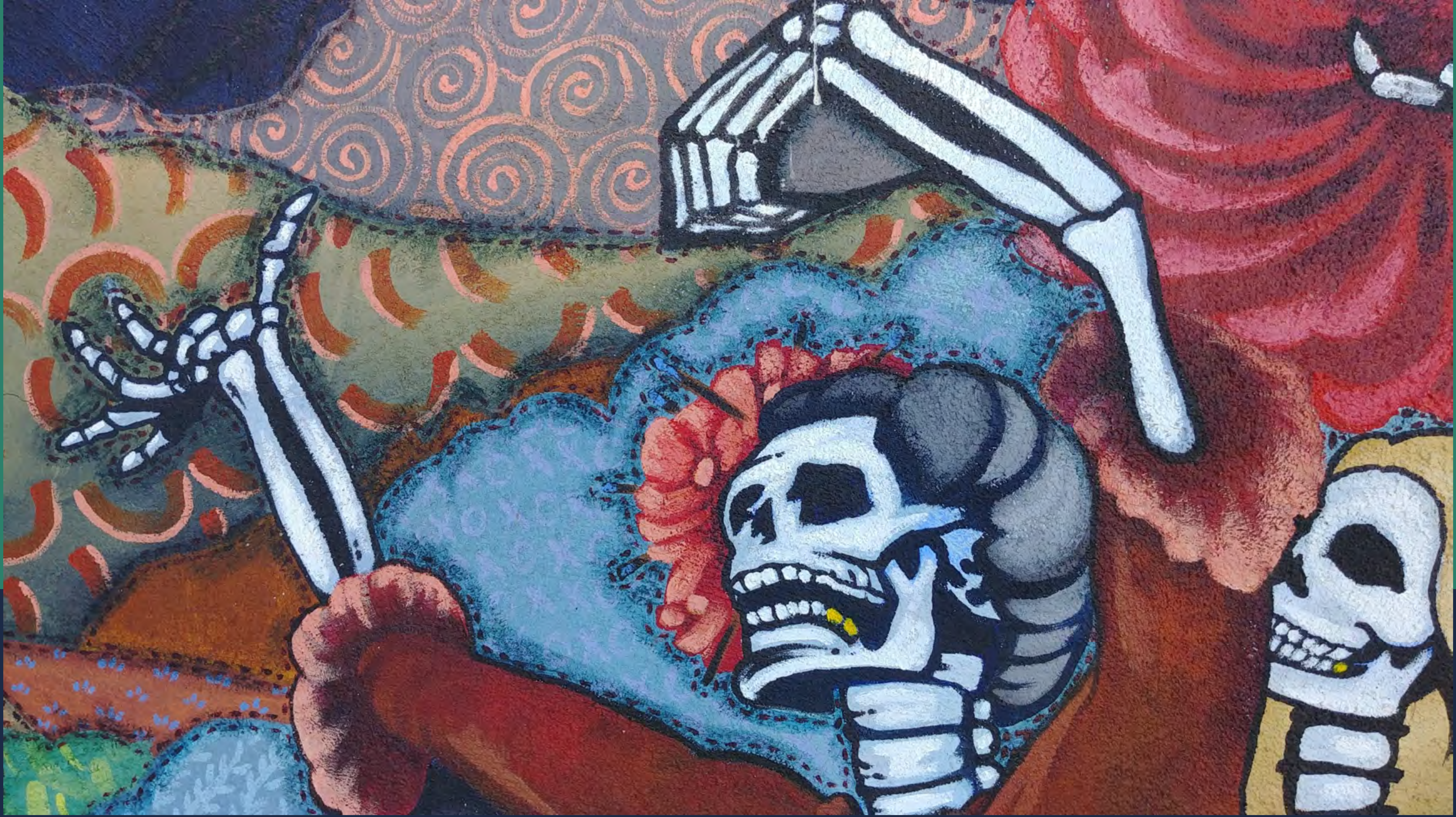

Dia de los Muertos mural, Why, Arizona

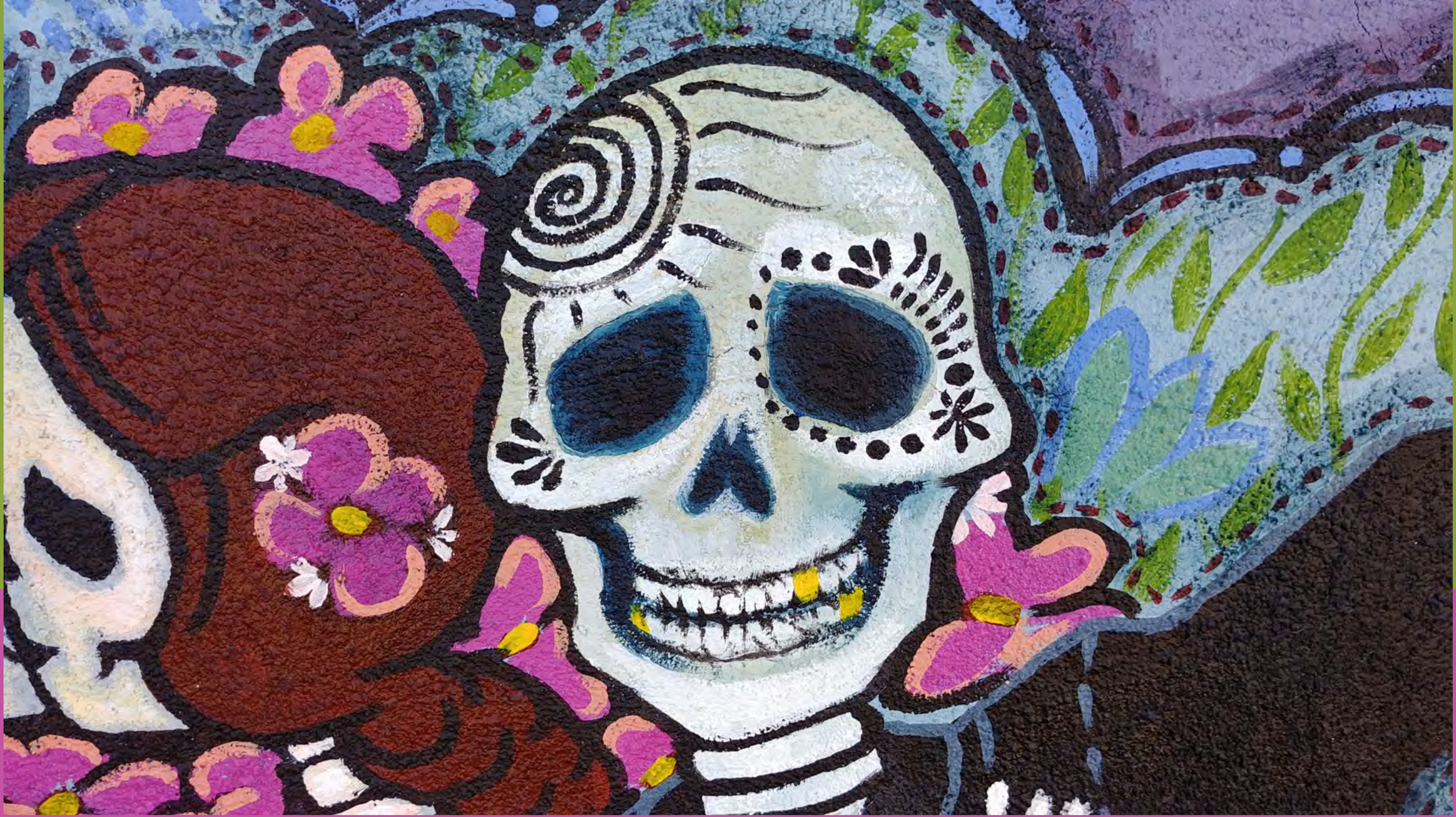

Dia de los Muertos mural, Why, Arizona

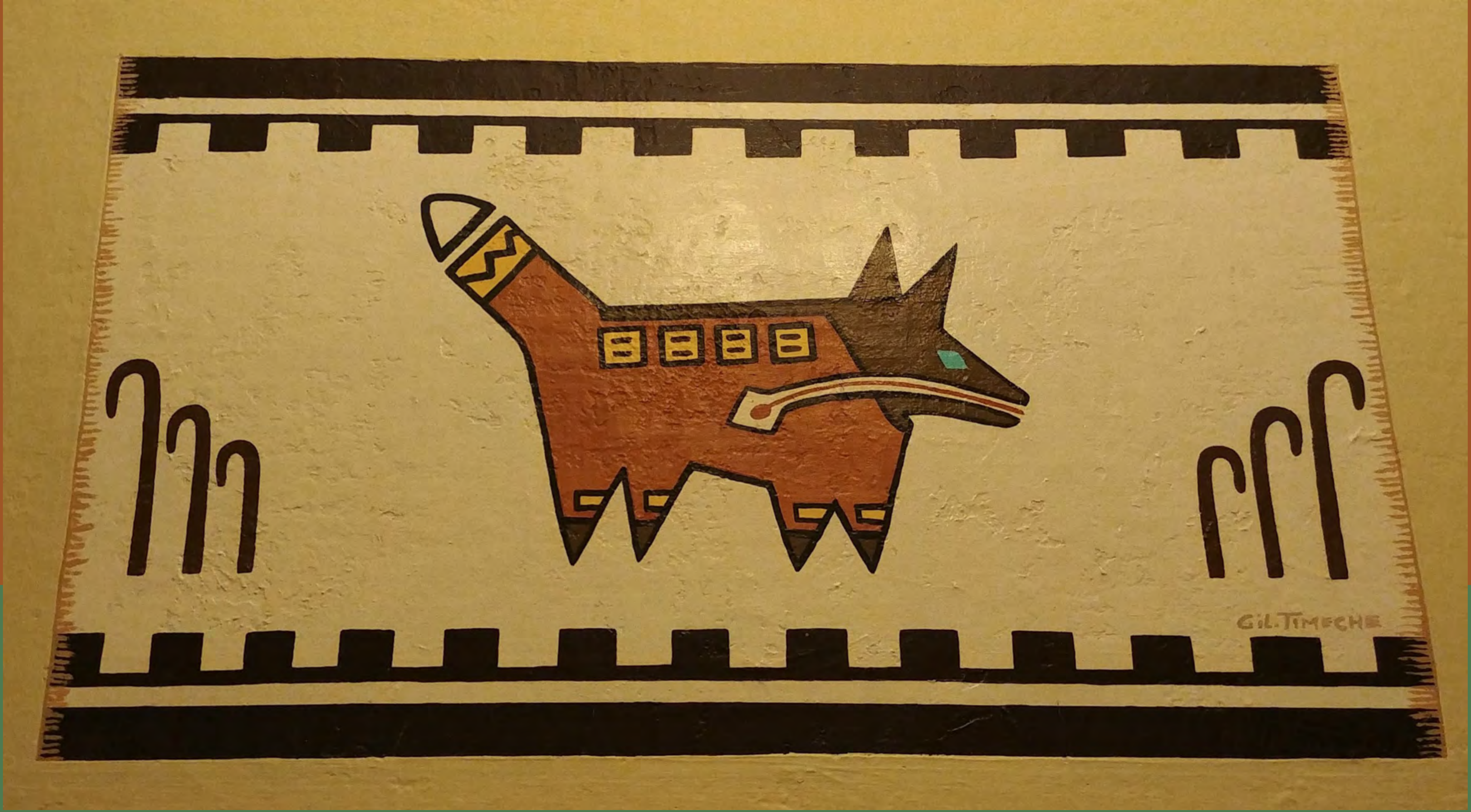


Native American motif, El Tovar Hotel, Grand Canyon

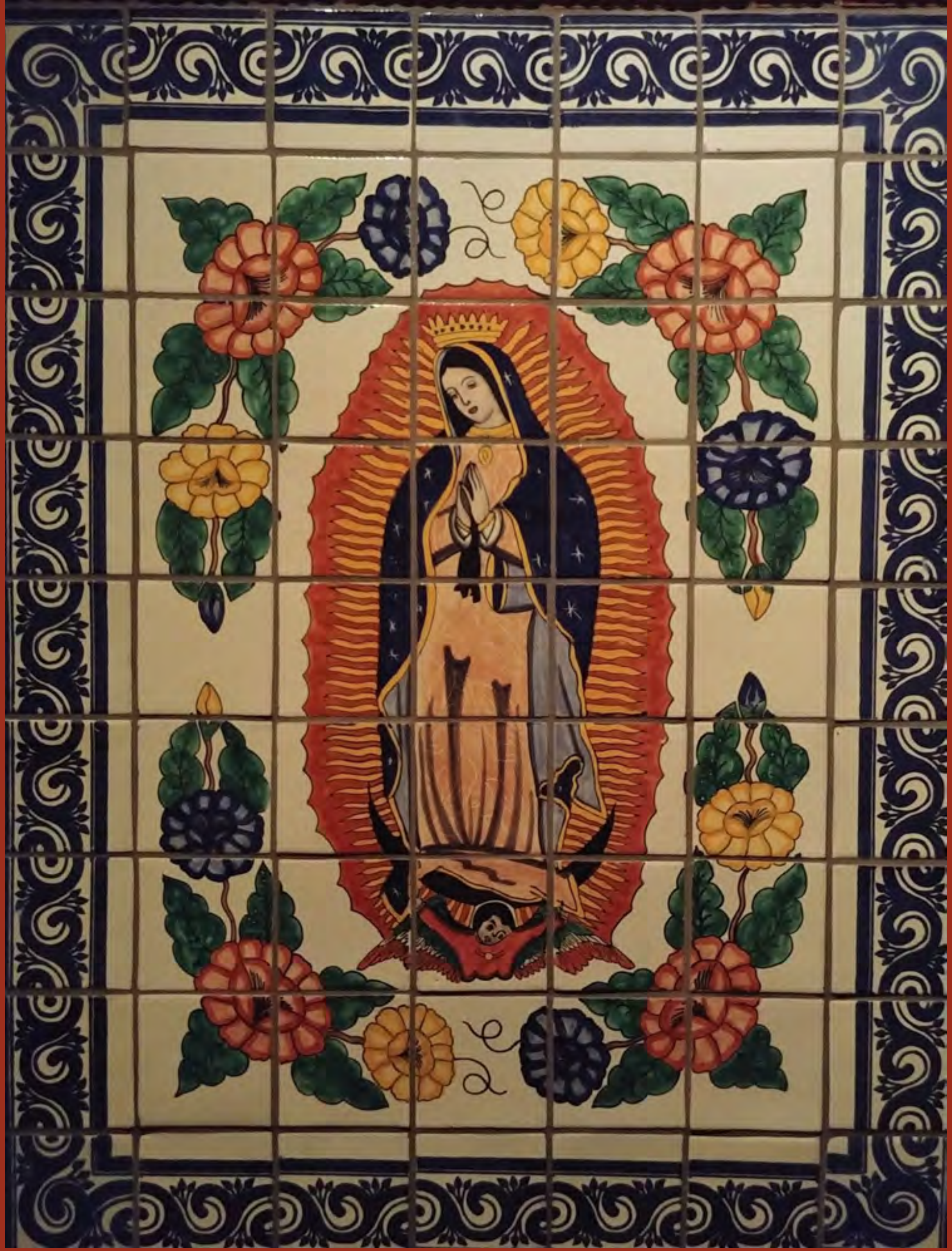

Virgen de Guadalupe, tile art, Tucson

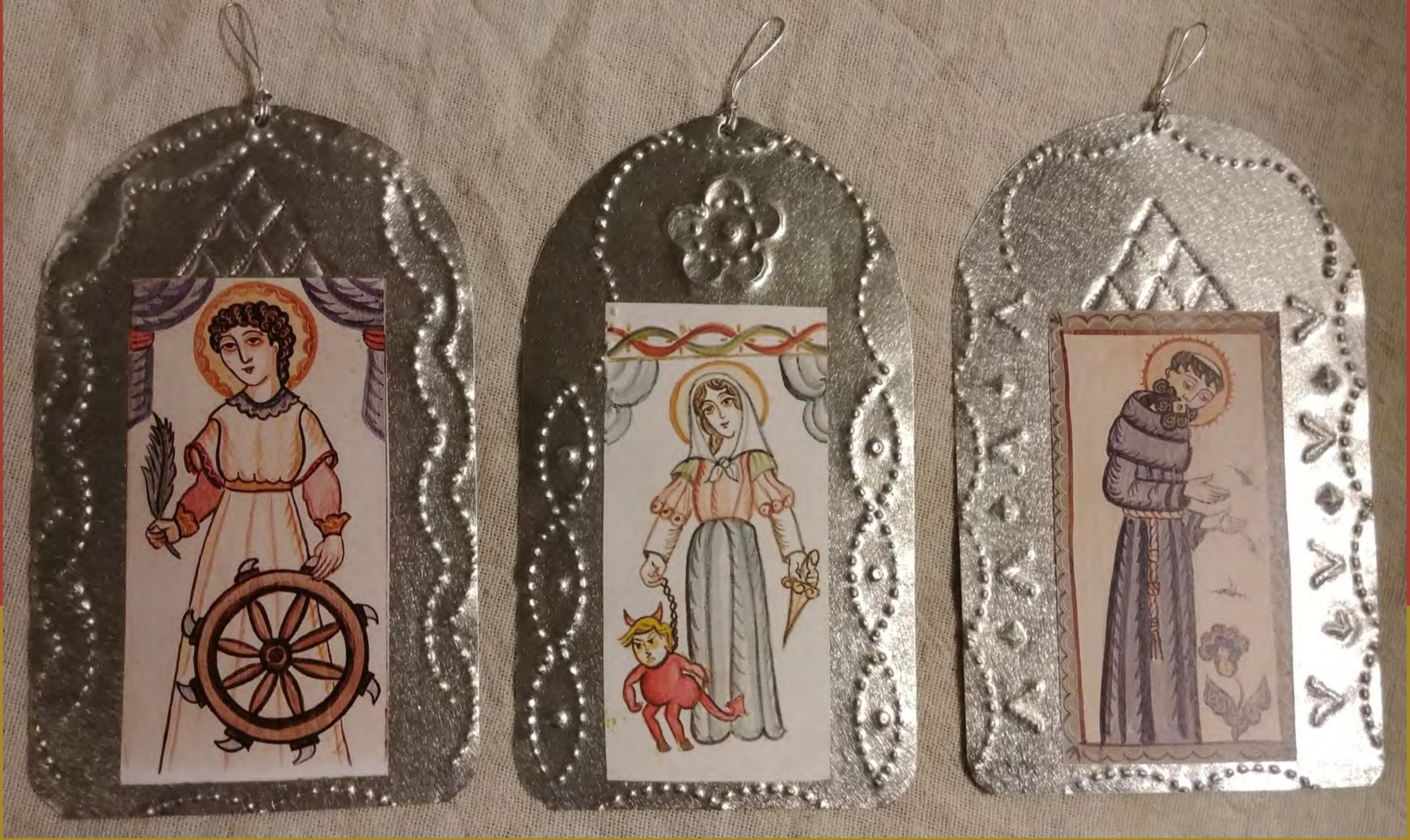

Santos, La Paloma, Tubac, Arizona

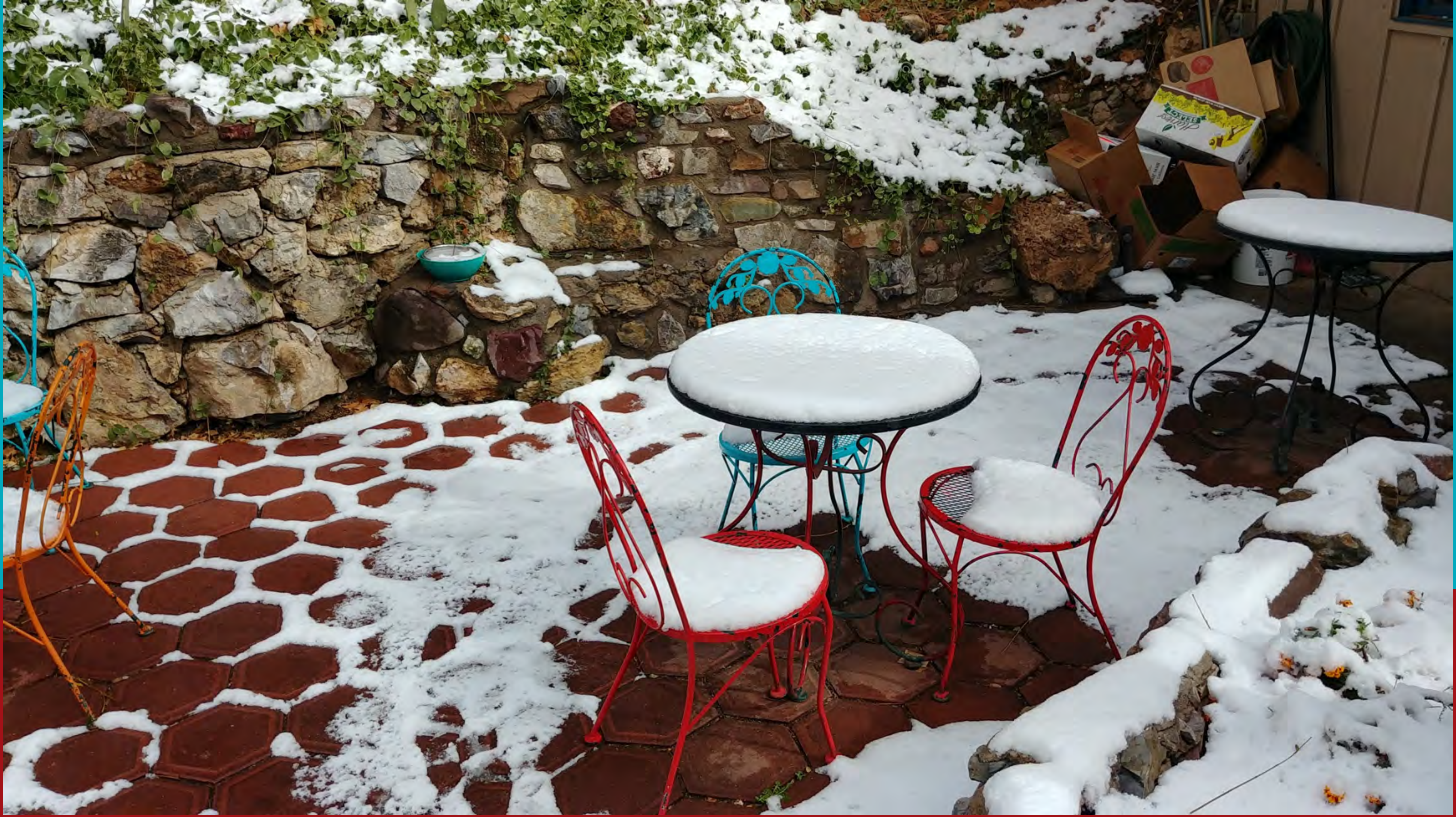

Bisbee in snow

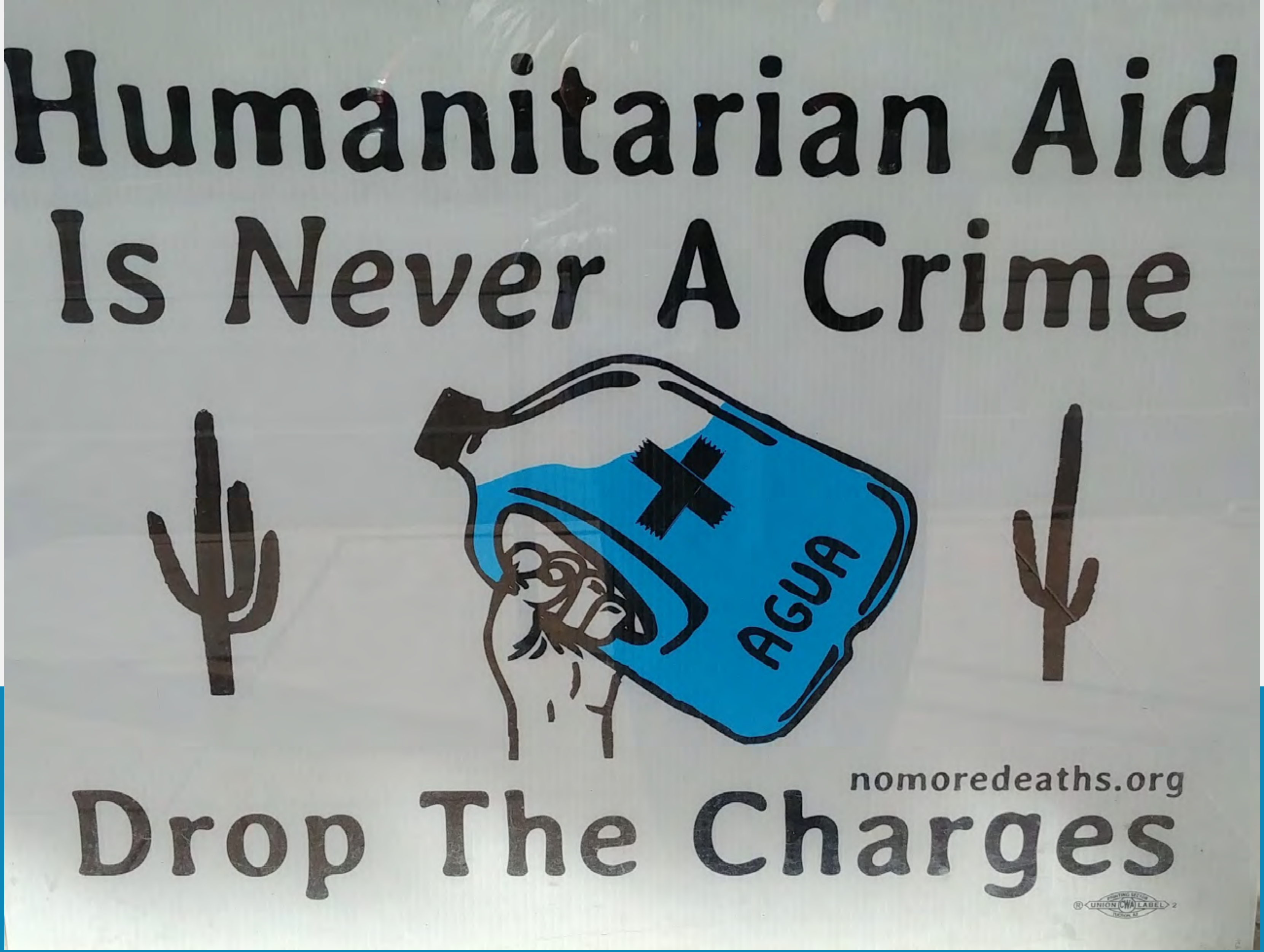


Tucson yard sign supporting Samaritan aid to desert border crossers

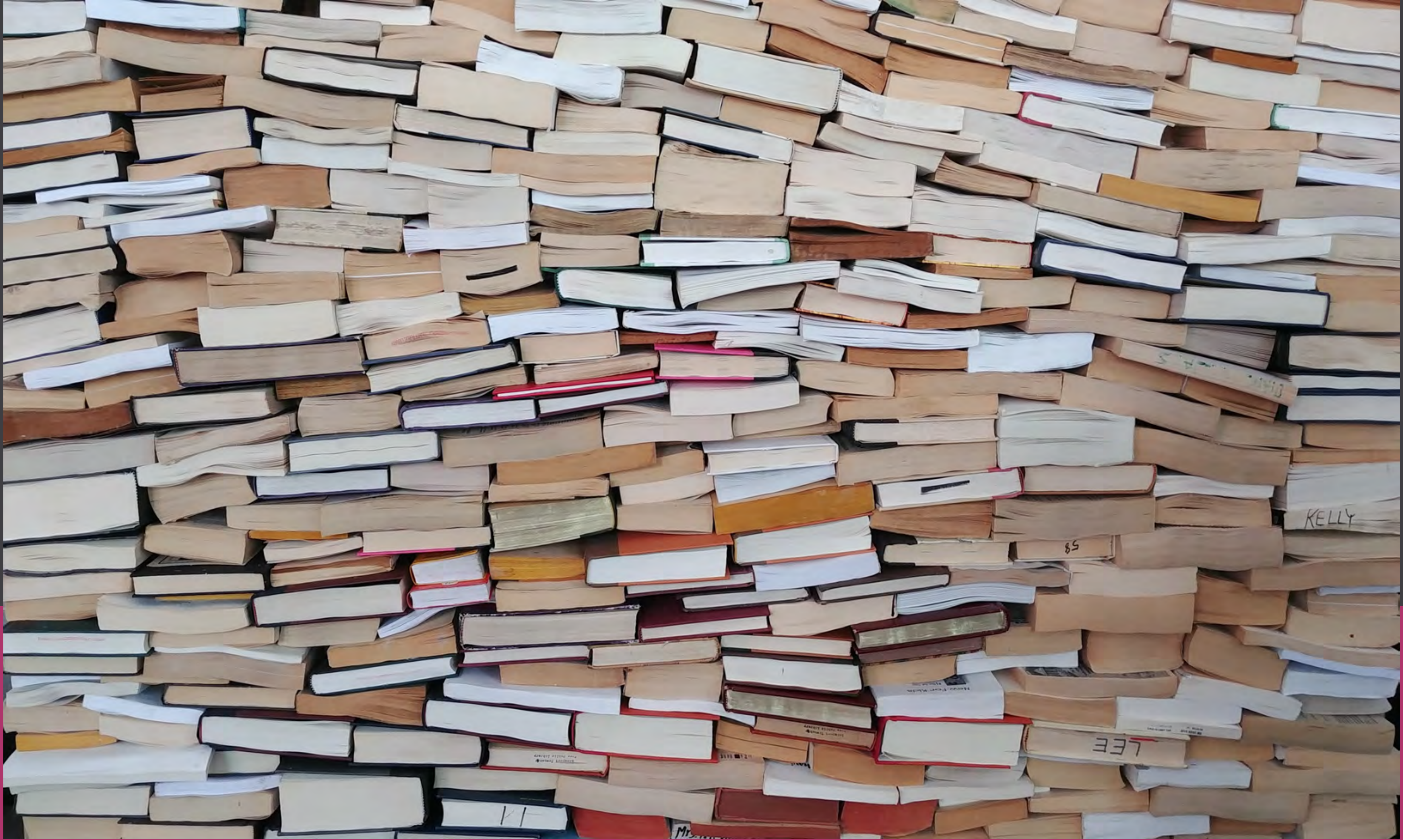


Wall of books

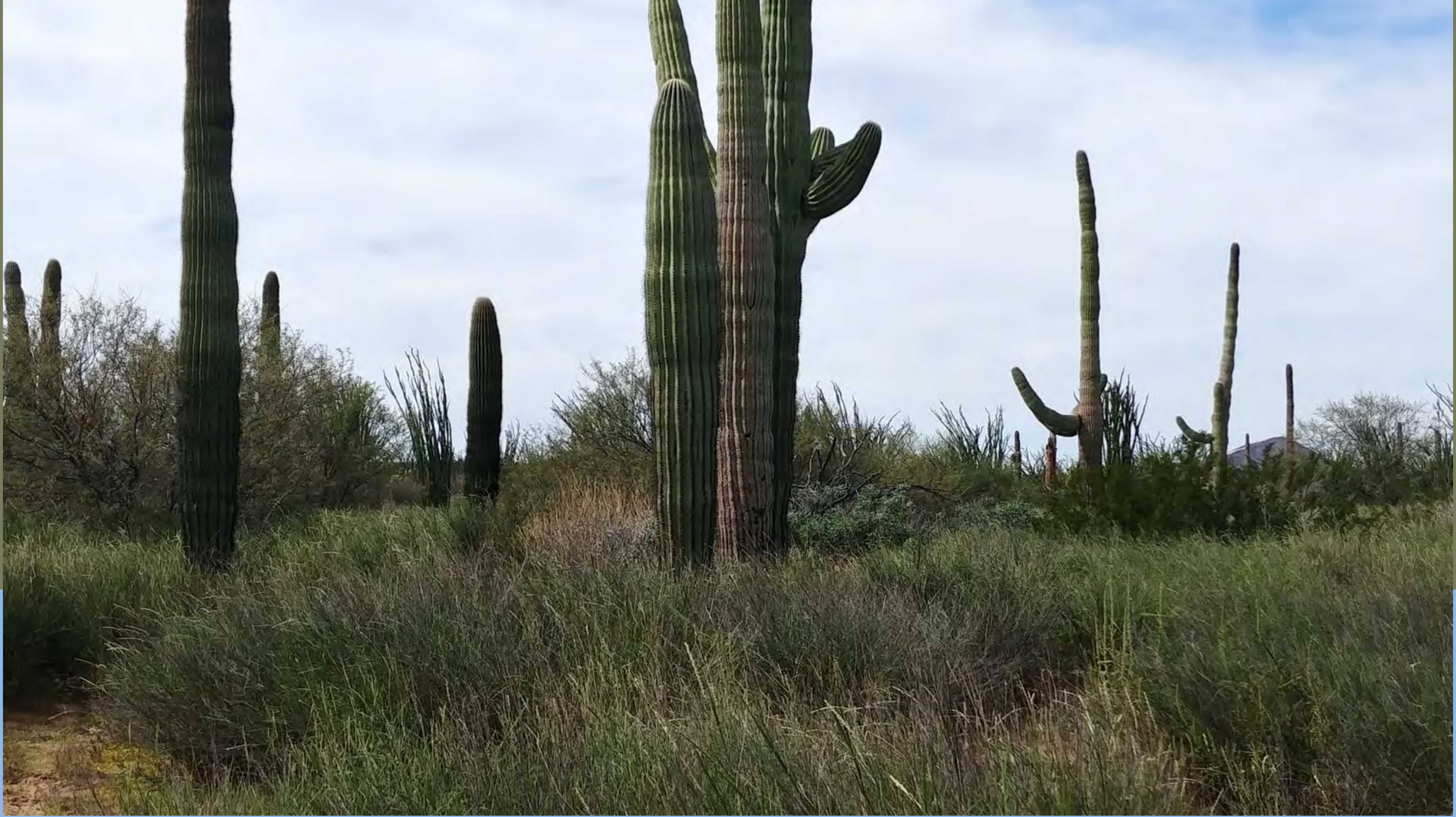

Sonoran Desert, Mexico/Arizona

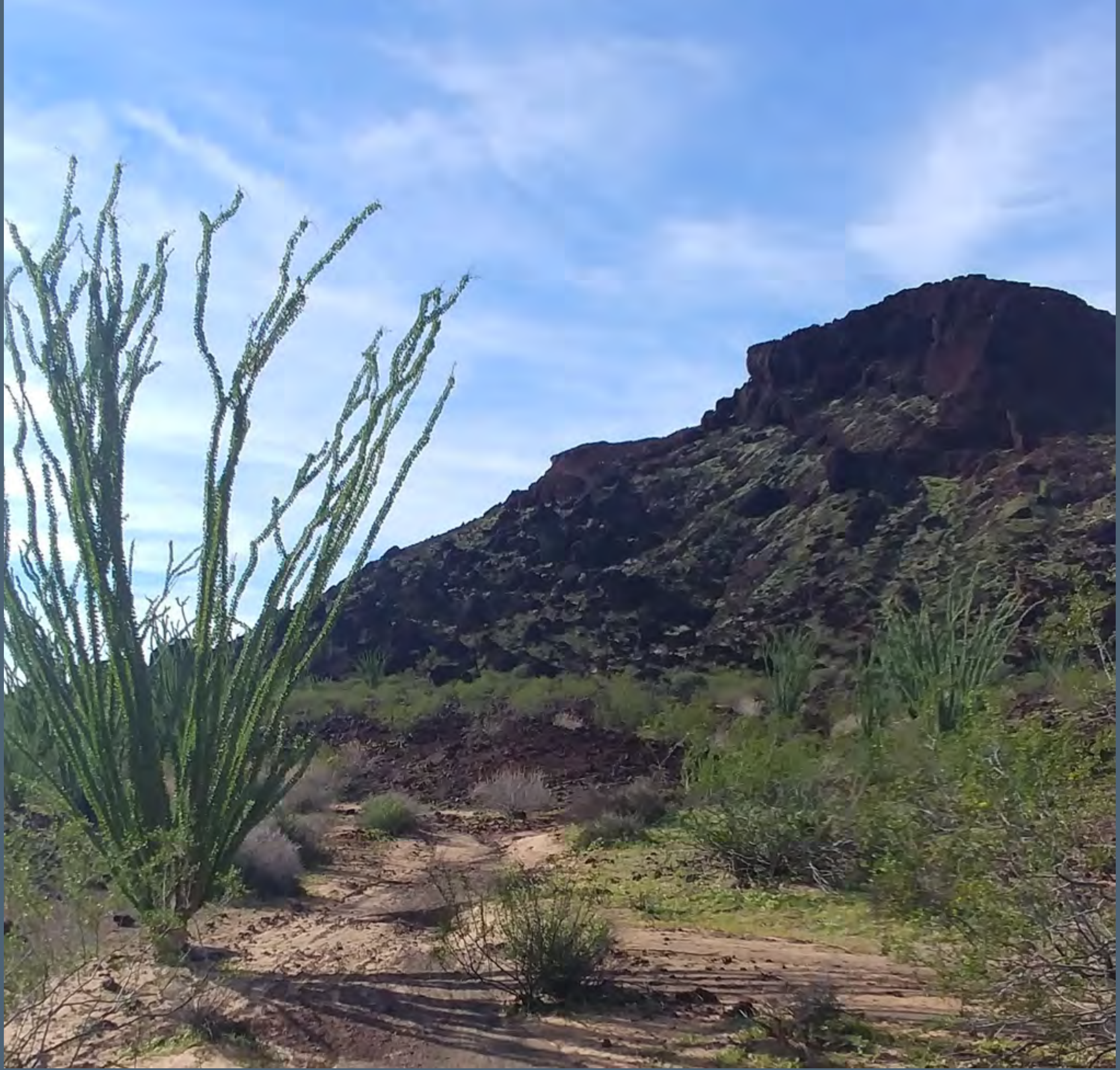

Pinecate, Sonora, Mexico

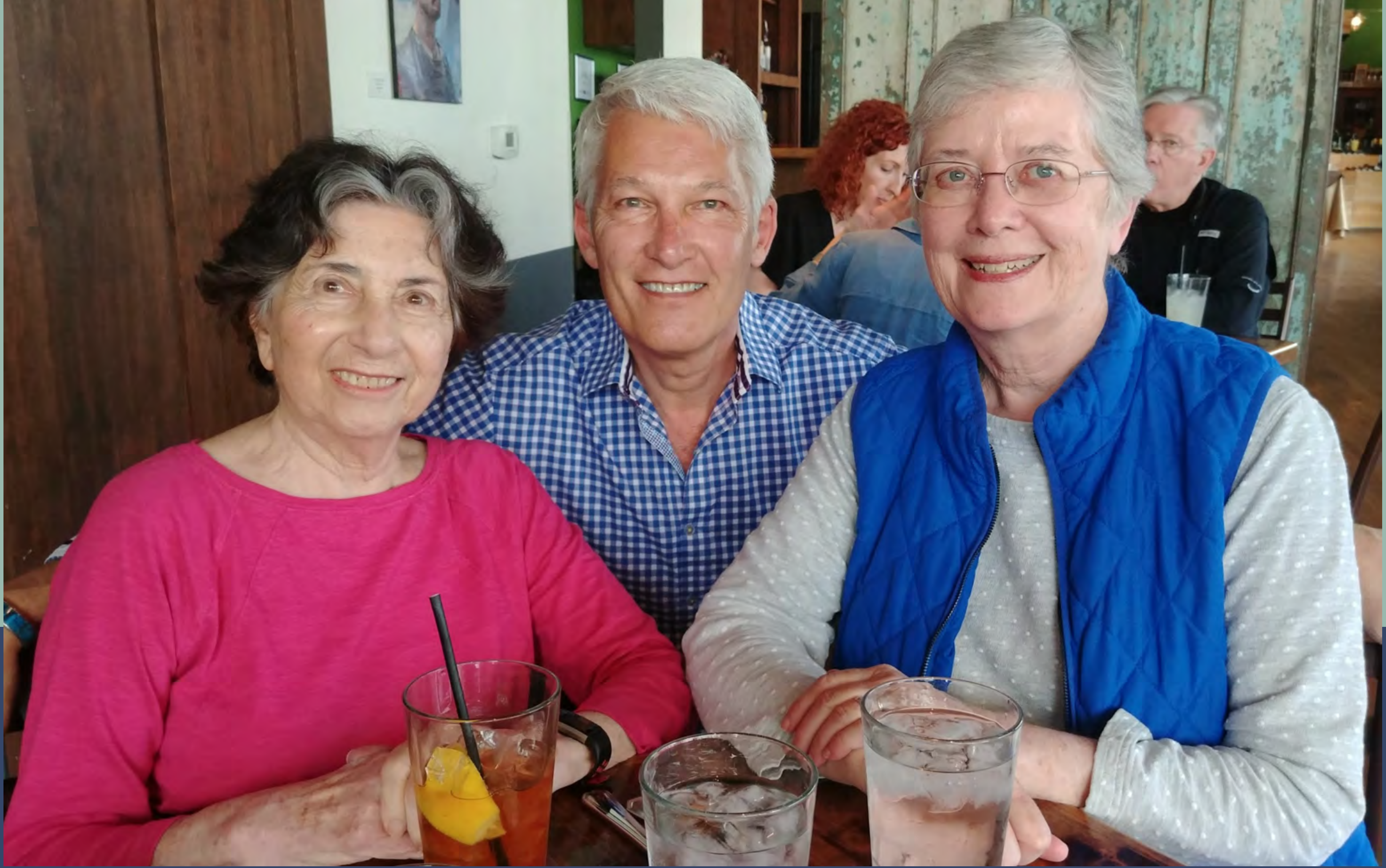

From left: Rachael K. Anderson, AHIP, FMLA, Doe Lecturer 1989; Rick B. Forsman, AHIP, FMLA, Doe Lecturer 2004; and Alison Bunting, AHIP, FMLA, Doe Lecturer, 1993

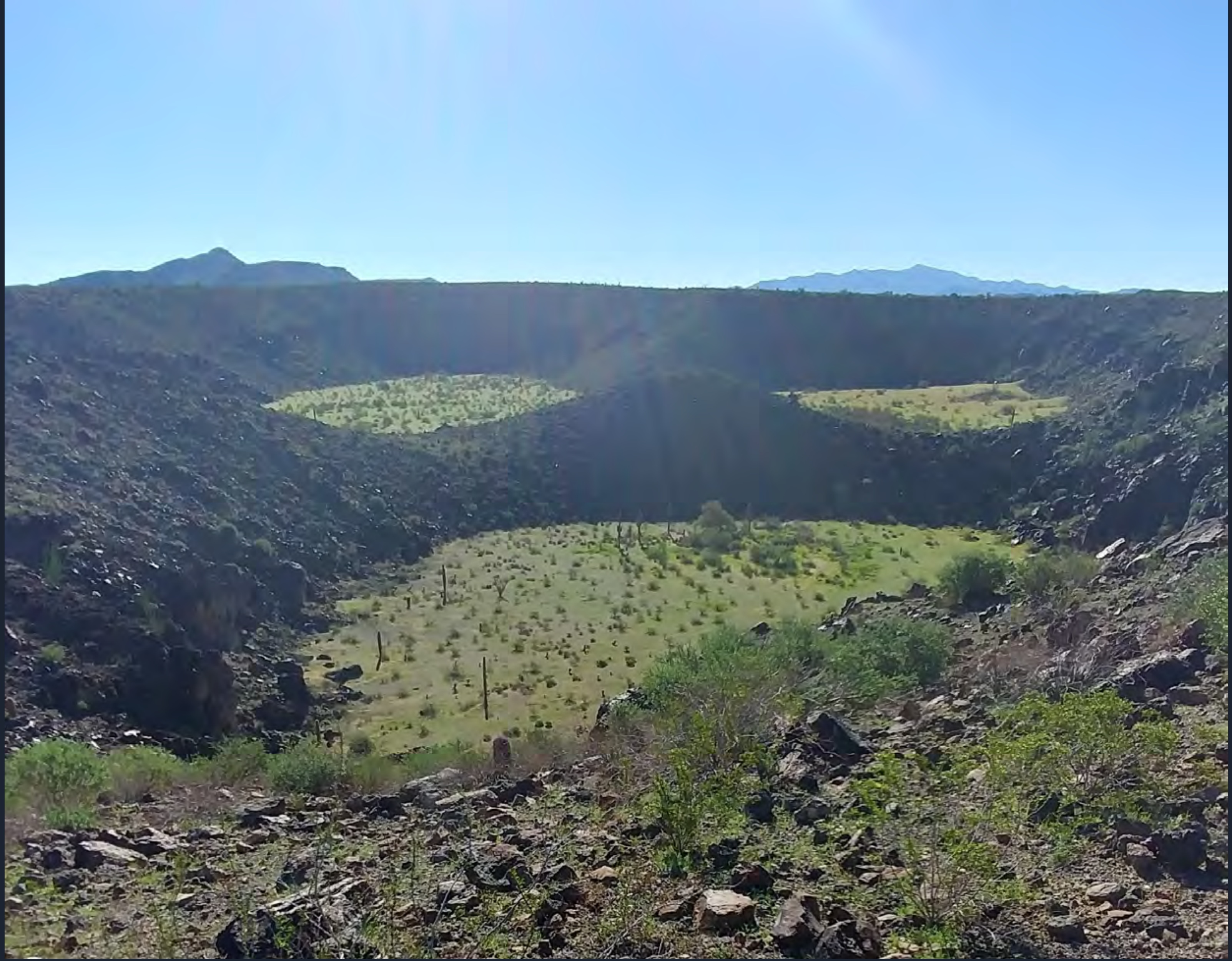

Pinecate, Sonora, Mexico

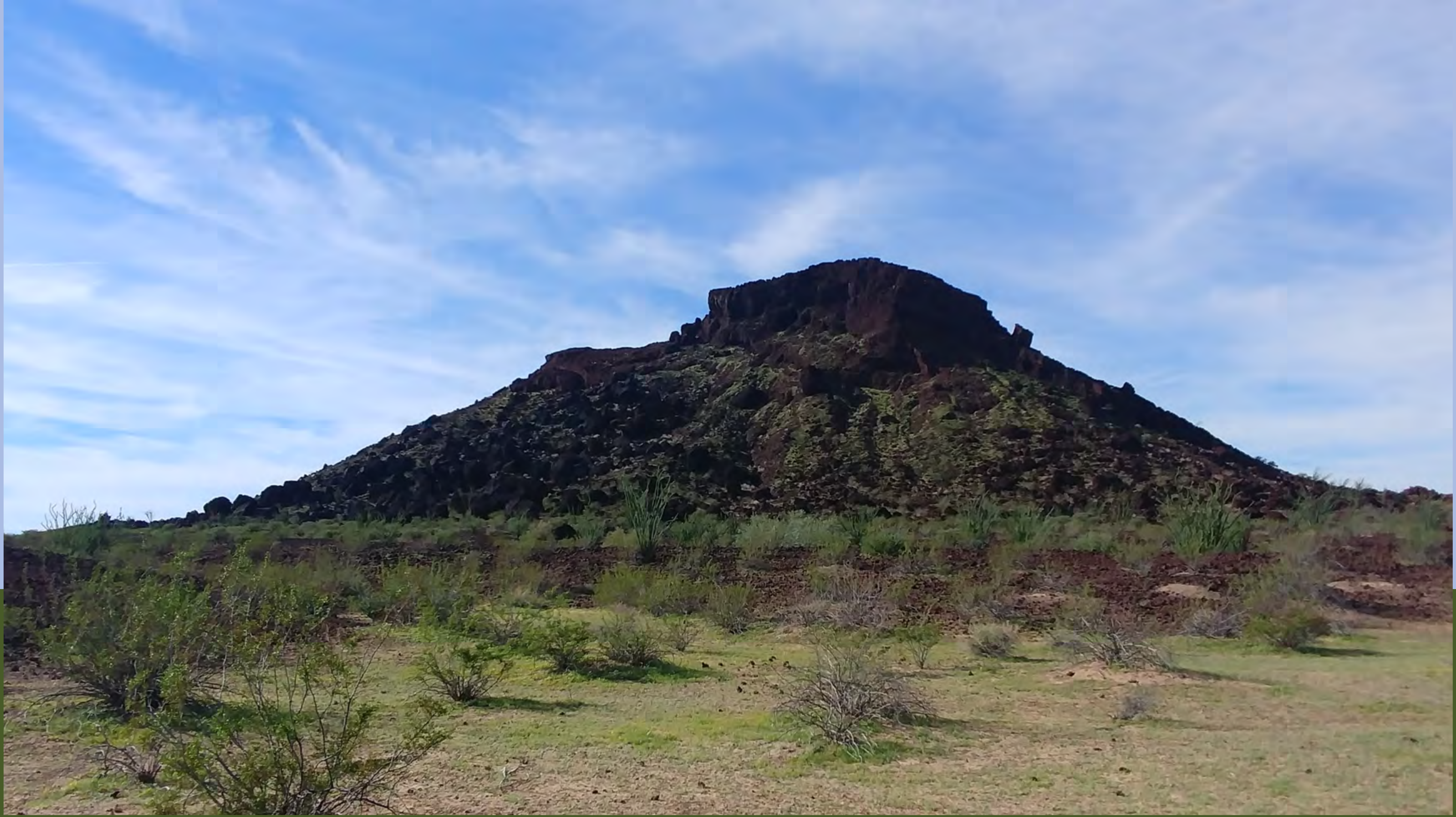

Pinecate, Sonora, Mexico

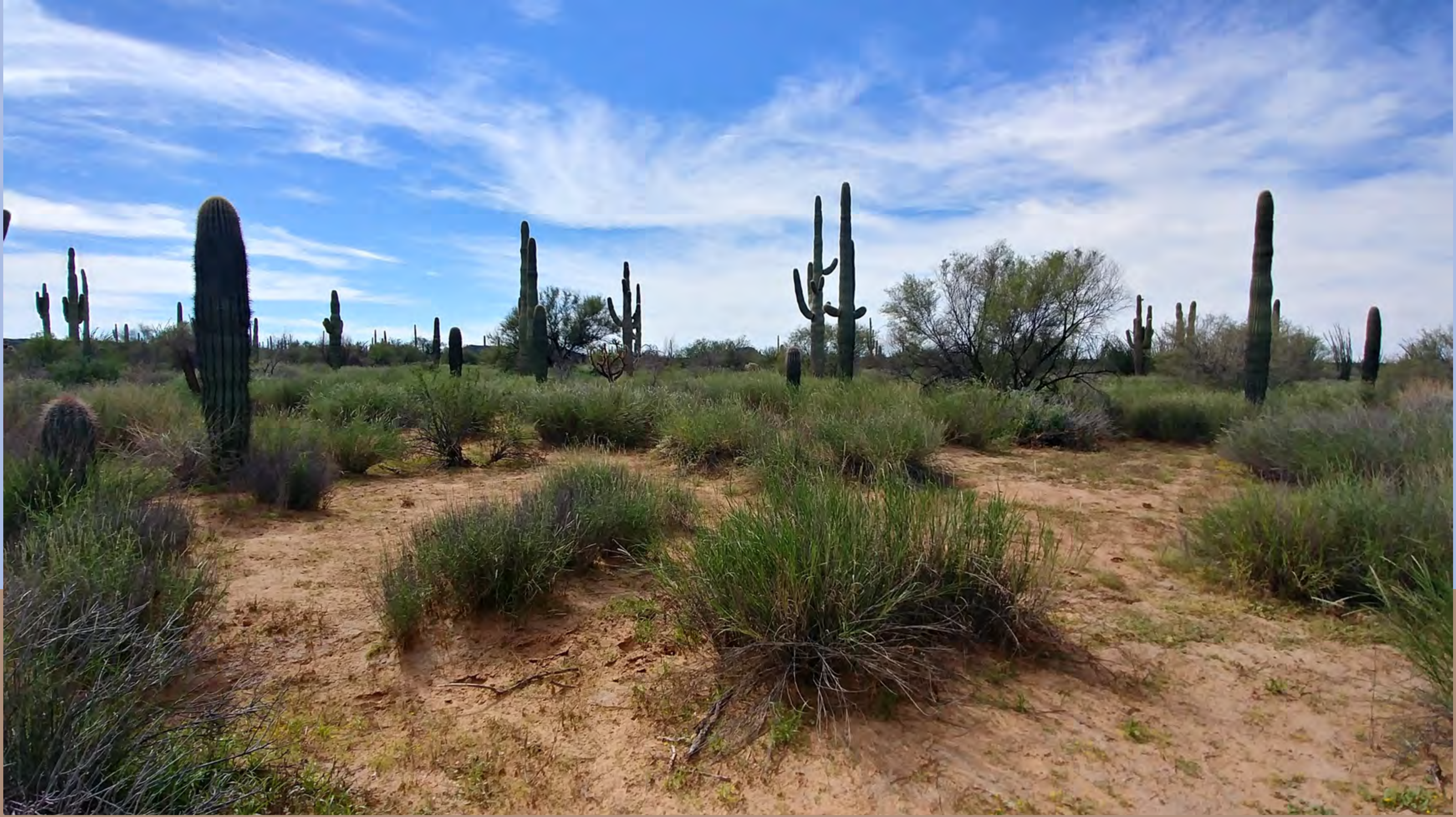

Sonoran Desert, Mexico/Arizona

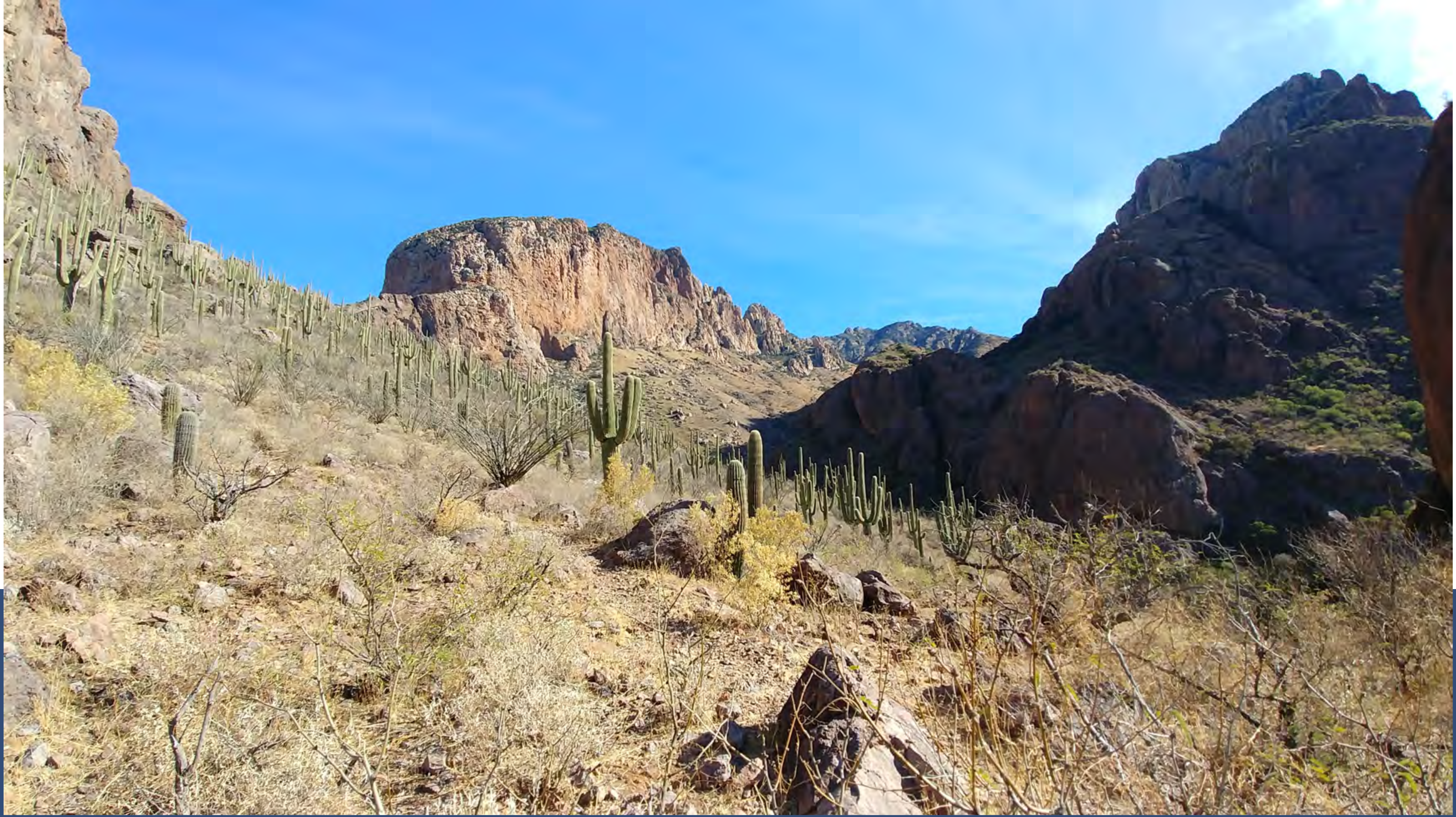

Tohono O'Oodham Reservation, Southern Arizona

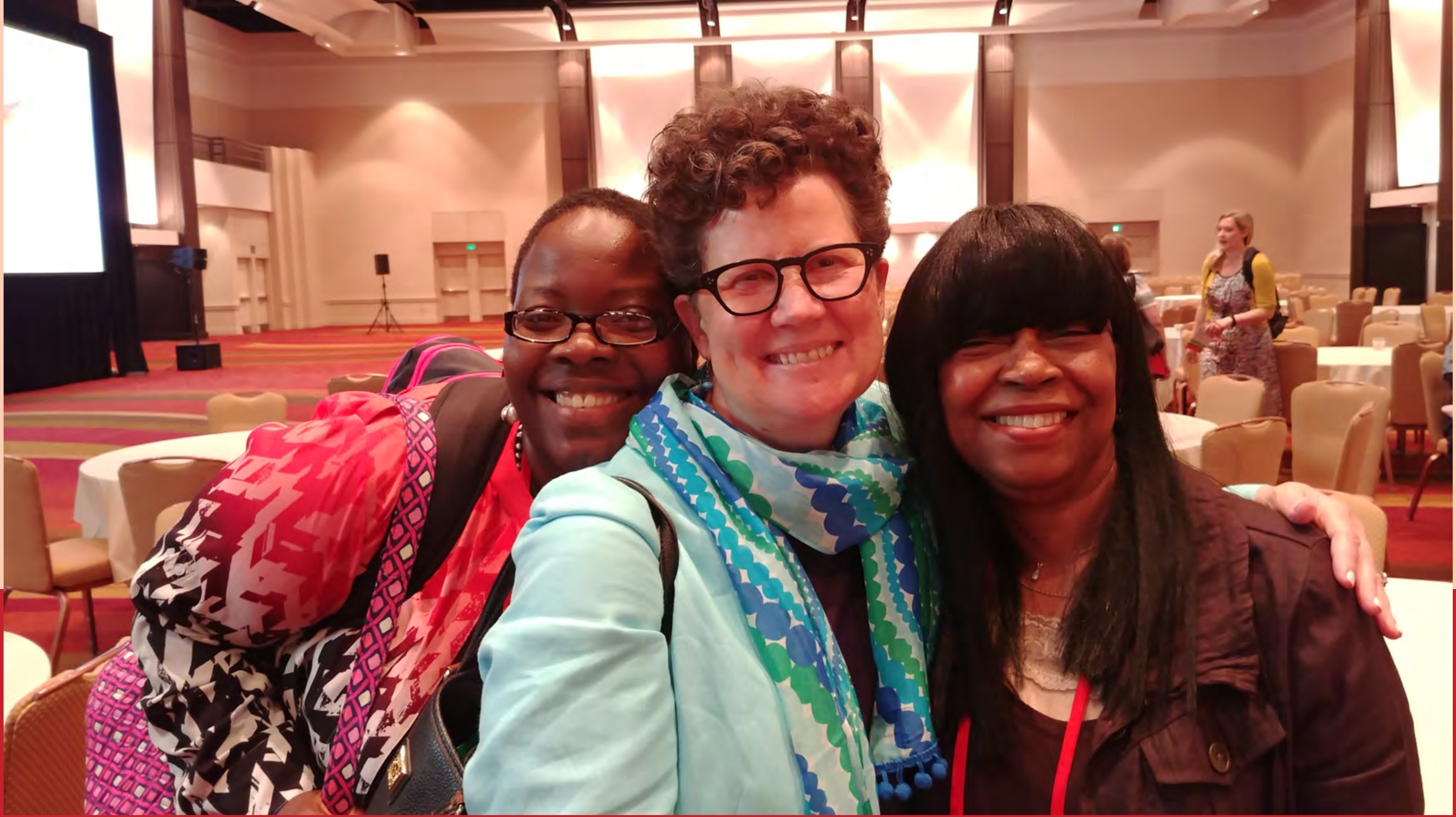

2018/19 President Beverly Murphy, AHIP, FMLA (right), with Shannon D. Jones, AHIP, and Ruth A. Riley, AHIP, at MLA ’18

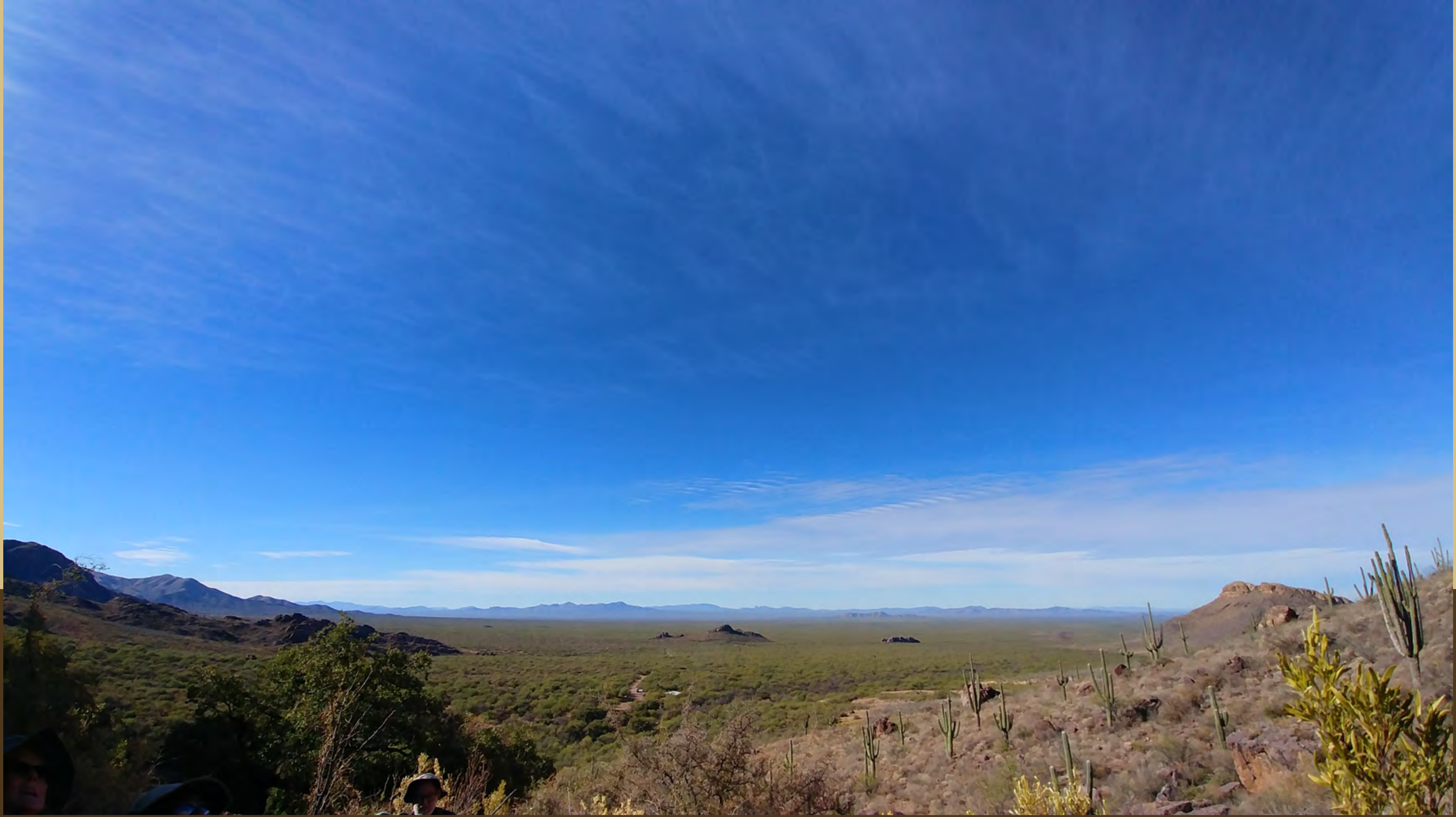

Tohono O'Oodham Reservation, Southern Arizona

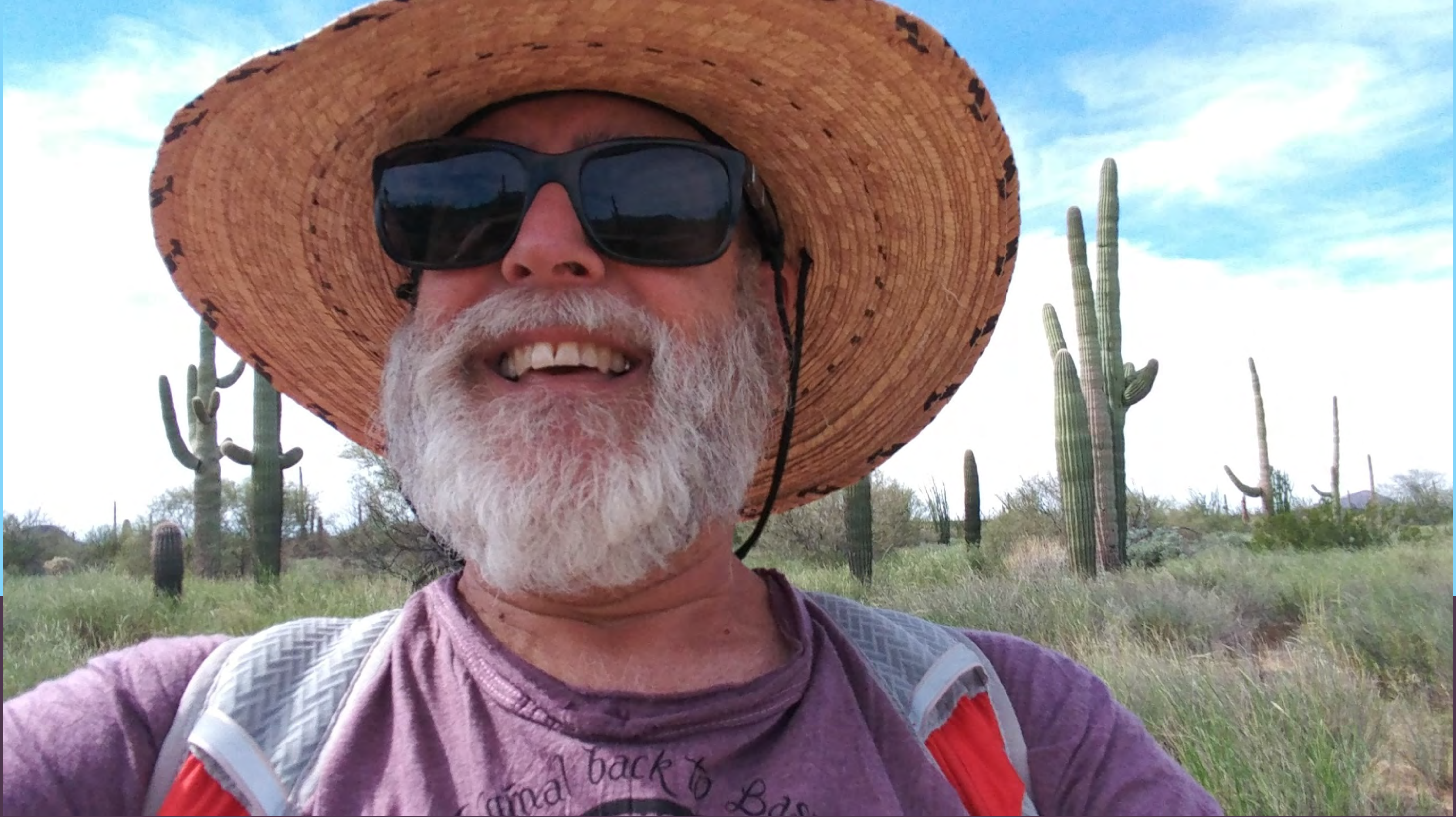

Field trip to the Pinecate, Sonora, Mexico

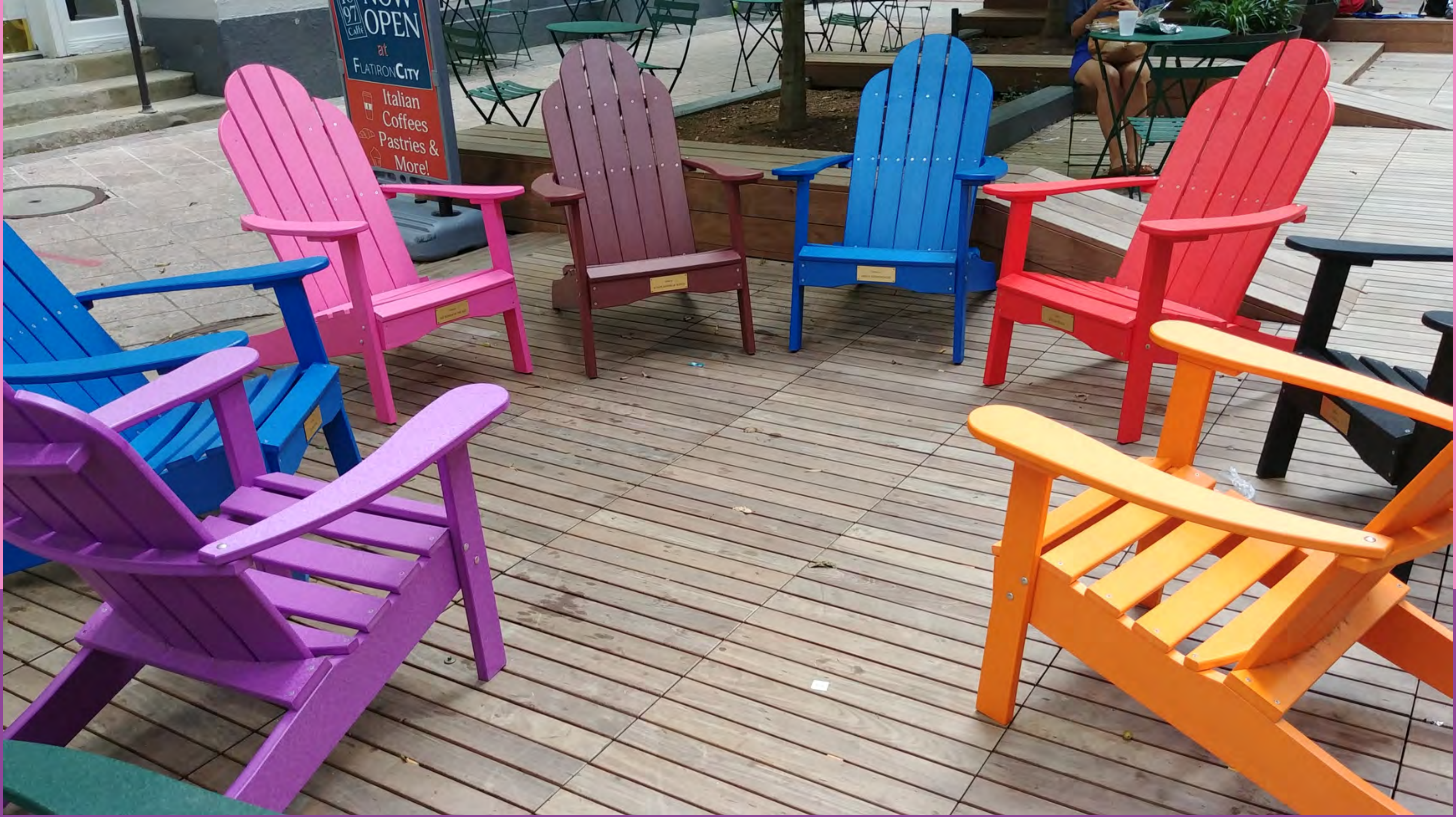


“Everybody’s Welcome” rainbow chairs

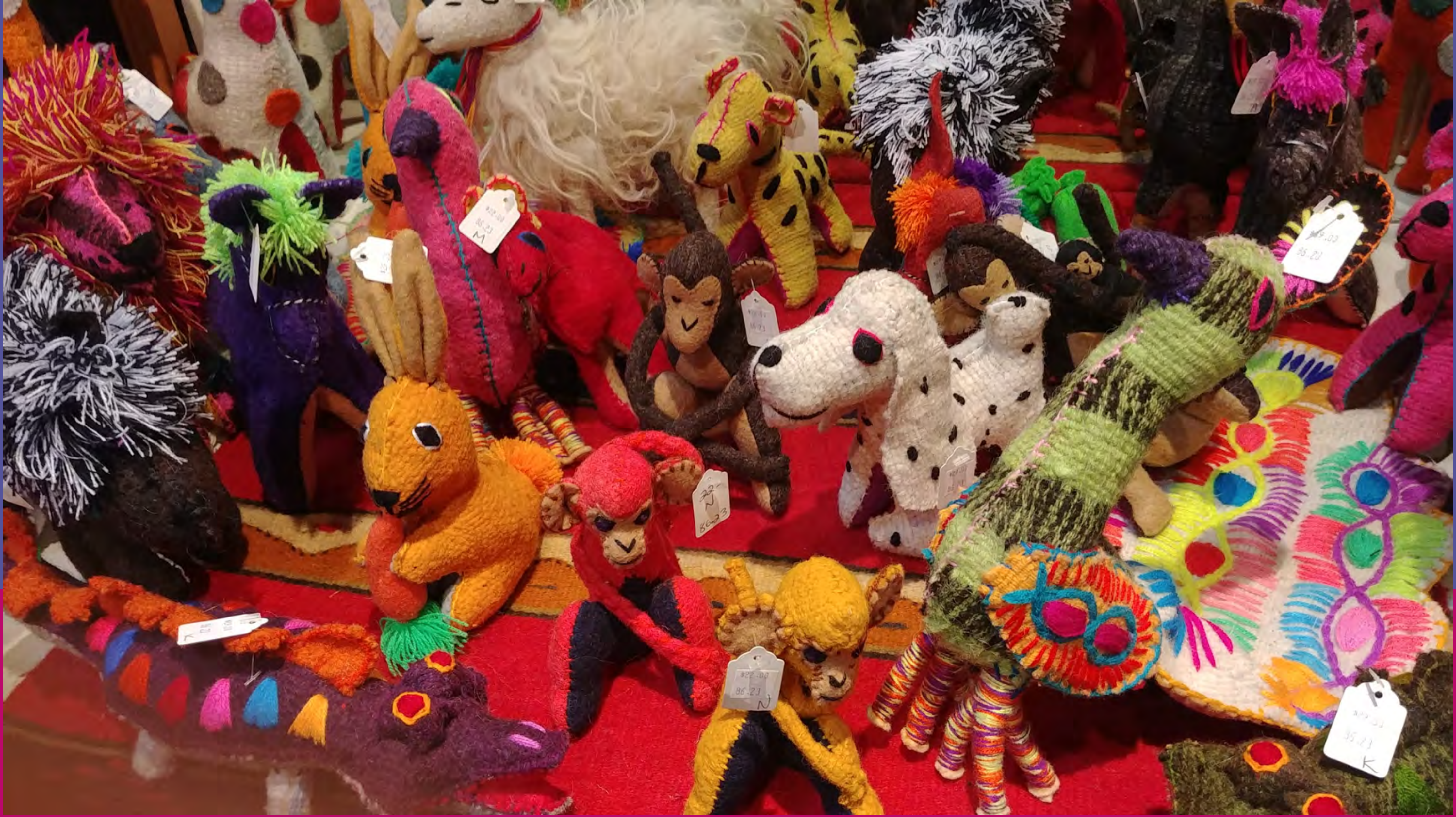

Toys, La Paloma, Tubac, Arizona

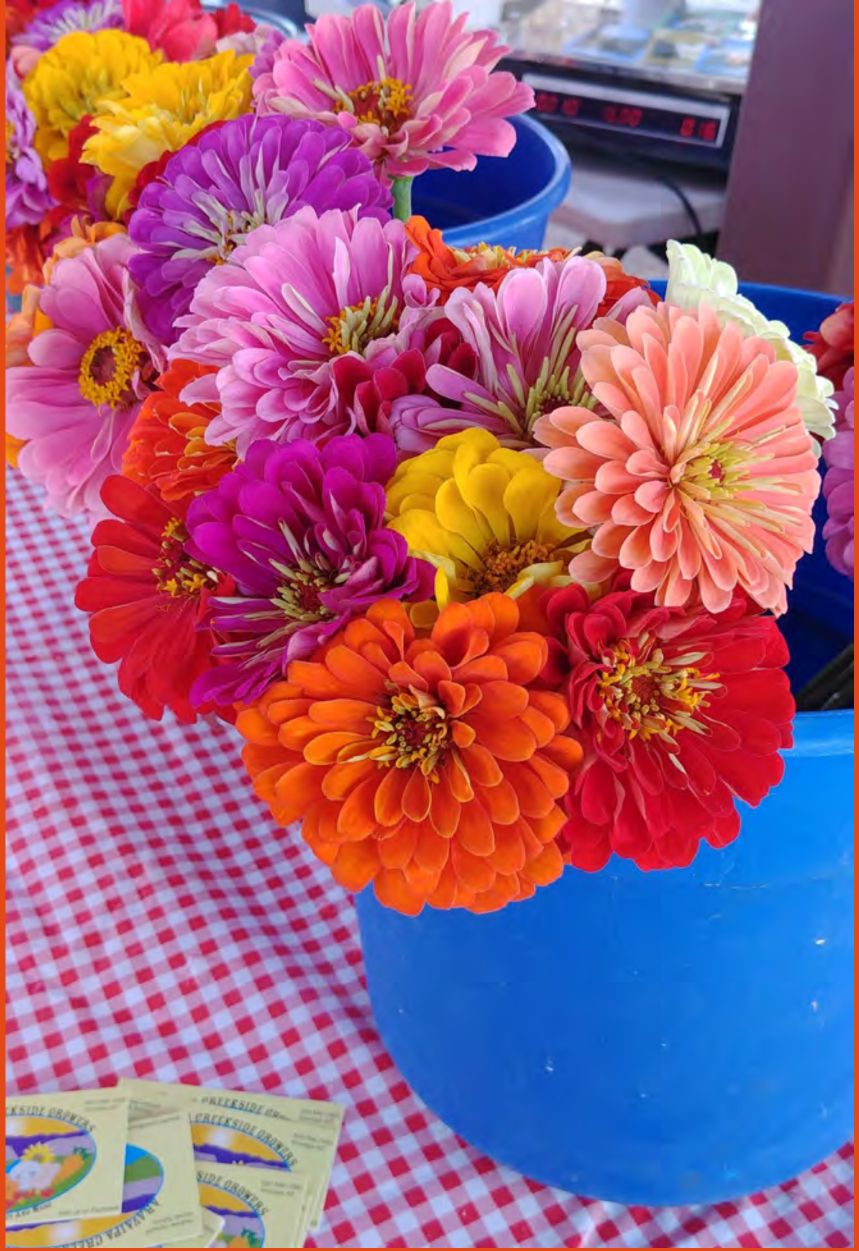


Zinnias, Farmer Market, Tucson

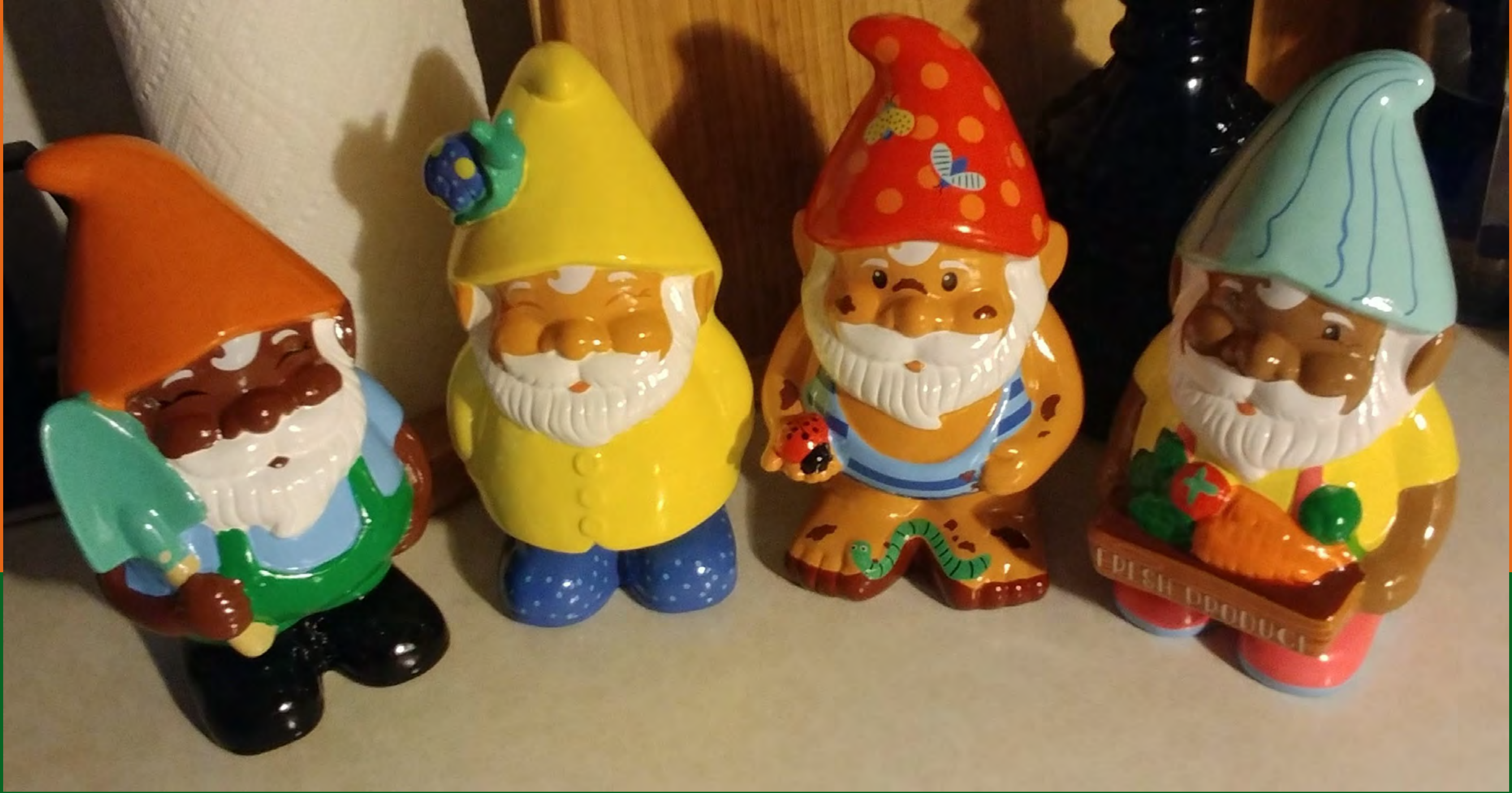


My garden gnomes

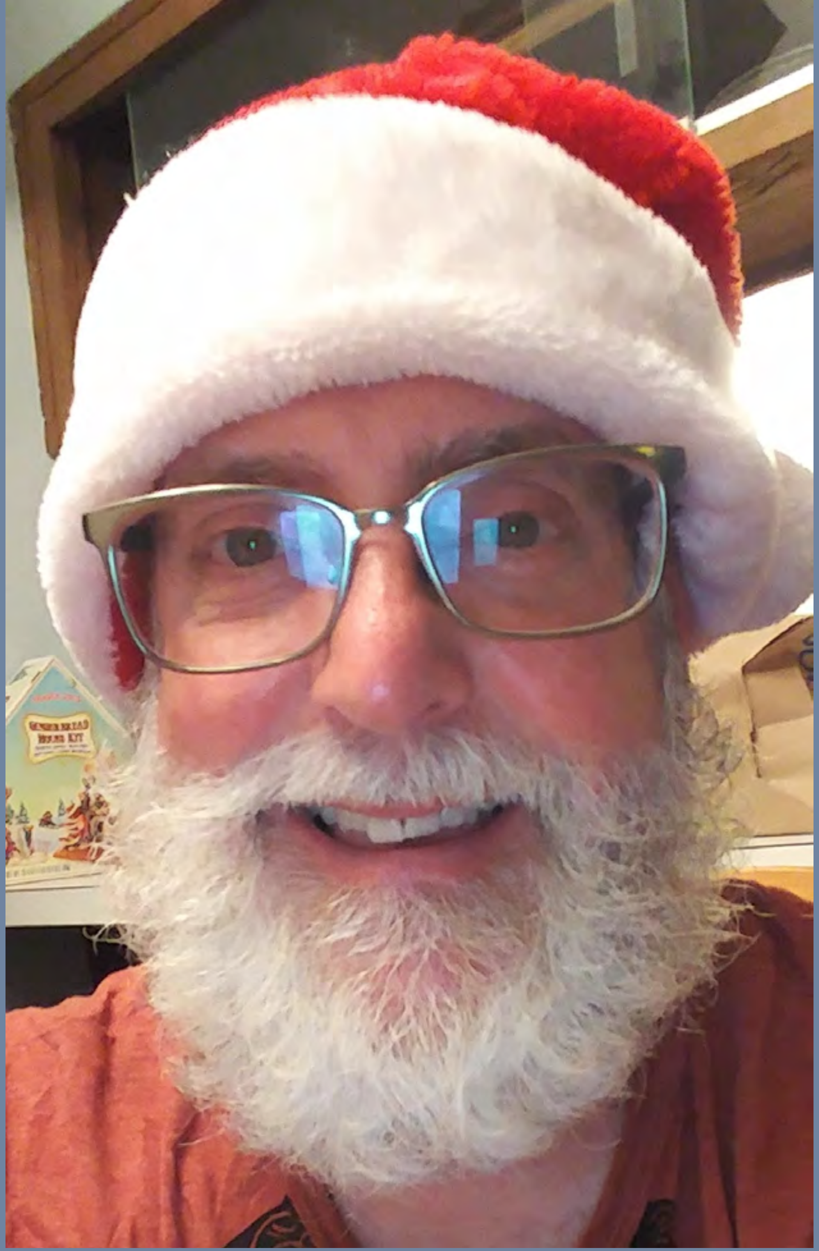

Thank You!!!
